# The elucidation of stress memory inheritance in *Brassica rapa* plants

**DOI:** 10.3389/fpls.2015.00005

**Published:** 2015-01-21

**Authors:** Andriy Bilichak, Yaroslav Ilnytskyy, Rafal Wóycicki, Nina Kepeshchuk, Dawson Fogen, Igor Kovalchuk

**Affiliations:** ^1^Lethbridge Research Centre, Agriculture and Agri-Food CanadaLethbridge, AB, Canada; ^2^Department of Biological Sciences, University of LethbridgeLethbridge, AB, Canada

**Keywords:** epigenetics, small RNAs, transcriptome, *B. rapa*, transgenerational inheritance

## Abstract

Plants are able to maintain the memory of stress exposure throughout their ontogenesis and faithfully propagate it into the next generation. Recent evidence argues for the epigenetic nature of this phenomenon. Small RNAs (smRNAs) are one of the vital epigenetic factors because they can both affect gene expression at the place of their generation and maintain non-cell-autonomous gene regulation. Here, we have made an attempt to decipher the contribution of smRNAs to the heat-shock-induced transgenerational inheritance in *Brassica rapa* plants using sequencing technology. To do this, we have generated comprehensive profiles of a transcriptome and a small RNAome (smRNAome) from somatic and reproductive tissues of stressed plants and their untreated progeny. We have demonstrated that the highest tissue-specific alterations in the transcriptome and smRNAome profile are detected in tissues that were not directly exposed to stress, namely, in the endosperm and pollen. Importantly, we have revealed that the progeny of stressed plants exhibit the highest fluctuations at the smRNAome level but not at the transcriptome level. Additionally, we have uncovered the existence of heat-inducible and transgenerationally transmitted tRNA-derived small RNA fragments in plants. Finally, we suggest that miR168 and *braAGO1* are involved in the stress-induced transgenerational inheritance in plants.

## Introduction

Plants constantly interact with environmental factors that can either benefit or jeopardize their homeostasis depending on the intensity and nature of factors encountered. Significant environmental perturbations that exceed the optimum range of plant development can cause stress and trigger the onset of gene expression changes in plants. Stress-induced alterations in the transcriptome profile have been shown to be both stress- and tissue-specific, although the general stress response (GSR) that has been extensively studied in yeast and animals is also present in plants (Kreps et al., 2002; Kultz, 2005; Dinneny et al., 2008; Walley and Dehesh, 2010; Iyer-Pascuzzi et al., 2011). Previously, we demonstrated that the progeny of plants exposed to salt stress and *Tobacco mosaic virus* (TMV) exhibit a higher tolerance not only to the same stressor but also to methyl methane sulfonate, a genotoxic agent that induces DNA methylation, as compared to control progeny (Boyko et al., 2010; Kathiria et al., 2010). Hence, although plants may trigger immediate specific gene expression changes to cope with a particular stressor, transgenerational inheritance and responses to stress seem to involve non-specific priming of stress-responsive genes. Distinct epigenetic mediators of multigenerational inheritance of stress memory have been recently identified in two animal models—*Drosophila* (Seong et al., 2011) and *C. elegans* (Buckley et al., 2012), which makes it tempting to argue for the existence of similar transgenerational mediators in plants.

Plants belonging to the genus *Brassica* are predominantly annual cool-season economically important crops whose cultivation is acutely affected by elevated temperatures and drought (Hall, 2001; Yu et al., 2012). Heat shock (HS) stress can severely influence reproductive tissues of plants, which contributes to poor seed set yield (Zinn et al., 2010). On the other hand, the pre-treatment of plants under moderate HS conditions can protect them from an acute heat stress and provide a better stress tolerance, a phenomenon known as induced or acquired thermotolerance (Gurley, 2000). The expression of heat-shock proteins regulated by heat stress transcription factors is believed to mediate the heat stress response and acquired thermotolerance in plants (Kotak et al., 2007; Yu et al., 2012).

Curiously, the acclimation to abiotic factors and induced resistance to pathogens (Van Loon, 1997) can be propagated into the next generation, a phenomenon known as transgenerational acquired tolerance (Boyko et al., 2010; Holeski et al., 2012). Given a practical value of such response for its implementation in plant biotechnology and agriculture, there has been a remarkable interest in unraveling pathways involved in transgenerational acquired tolerance. Currently, two different mechanisms are proposed to account for the aforementioned phenomenon: maternal effects on seed size (Agrawal, 2001) and epigenetic alterations that allow a vertical propagation of acquired traits without changing the underlying genomic DNA sequence (Jablonka and Raz, 2009). Whereas the former mechanism has little support from experiments on plants subjected to multigenerational stress, the evidence favoring the second mechanism has been provided at the molecular level (Jablonka and Raz, 2009; Boyko et al., 2010; Bilichak et al., 2012; Luna et al., 2012; Rasmann et al., 2012; Slaughter et al., 2012).

Heritable fluctuations in DNA methylation, chromatin composition and smRNA metabolism are among the primary causes of transgenerational epigenetic inheritance (Holeski et al., 2012). Being reversible in their nature, the acquired components of the epigenetic machinery are usually prone to significant alterations during sexual plant development. In angiosperms, epigenetic factors have to survive the multifaceted process of gametogenesis and early embryogenesis that encompasses a significant restructuring of both cells and chromatin (Ibarra et al., 2012). Although methylation at CpG sequences found in vegetative cells is largely retained in sperm cells, embryo and conceivably in egg cells, methylation at the asymmetric CpHpH (where H = A, C, or T) sequences is largely lost as compared to vegetative cells (Gehring et al., 2009; Hsieh et al., 2009b; Ibarra et al., 2012; Jullien et al., 2012). Curiously, in all three cases (in sperm, egg cell and embryo), asymmetric DNA methylation is proposed to be restored through the RNA-dependent DNA methylation (RdDM) pathway guided by smRNAs generated by companion cells or tissues that do not contribute genetic material to the progeny. In turn, smRNAs have been shown to be vital environmental sensors, the expression of which is acutely affected by abiotic and biotic stressors (Khraiwesh et al., 2012). Therefore, we hypothesized that perturbations in the expression of smRNAs in somatic tissues caused by environmental fluctuations would eventually be imprinted in the transcriptome patterns in gametes and progeny.

## Materials and methods

### Plant cultivation

In the current work, R-o-18 a rapid-cycling self-compatible inbred line of *Brassica rapa* var. *trilocularis* (Roxb.) Hanelt (yellow sarson) was used (Williams and Hill, 1986; Amoah et al., 2012). Seeds were originally obtained from Dr. Joan E. Krochko, the NRC Plant Biotechnology Institute (Saskatoon, Saskatchewan, Canada).

### Growing *B. rapa* plants for heat-stress experiments and tissue harvesting

Seeds obtained from a single unstressed plant were germinated on *All purpose potting soil* (Plant *Etc*; Lethbridge, AB, Canada) mixed in the proportion 4:1 with vermiculite (The Professional Gardener Co LTD, AB, Canada) in 4 × 4 inch square pots. The soil mixture was pre-soaked once with Miracle-Gro fertilizer (Scotts Canada *Ltd.*, Mississauga, ON, Canada) and was maintained constantly moist with tap water. Rapid-cycling *B. rapa* parental plants (30 plants per group) and their progeny were cultivated in biochambers (Biofoot^TM^, model GC-20, Winnipeg, MB, Canada) under continuous high-intensity cold light illumination (200 μmol m^−2^ s^−1^) provided by fluorescent lamps and a 60% relative humidity at 22°C as described previously (Daugherty and Musgrave, 1994; Tel-Zur and Goldman, 2007). The cultivation of plants under constant light did not affect their phenotypic appearance as compared to plants grown under a long-day photoperiod (16 h day, 8 h night). Two weeks post germination, before the appearance of apical inflorescence shoots, plants from the treated group were exposed to 42°C for 3 h per day for 7 days in a row (Supplementary Figure 1). Subsequently, plants from both control and treated groups and their progeny were grown in continuous light at 22°C.

In order to monitor the development of stress memory and follow its transmission to germ cells, 17 days after heat-shock treatment, middle leaves and up to 1 cm of the inflorescence meristem (32 days post germination) dissected from flower buds of control and stressed plants were harvested.

To follow the occurrence of epigenetic marks in reproductive tissues, the pollen and unfertilized and fertilized ovules from both groups were harvested separately. The inflorescences were covered with plastic bags to prevent cross-pollination between treatment groups. The pollen from control and heat-shock treated plants was harvested separately using a vacuum manifold method (Johnson-Brousseau and McCormick, 2004).

The unfertilized ovules containing mature embryo sacs were synchronized developmentally by emasculating flowers at stage 13 (Smyth et al., 1990). Twenty four hours later, the ovules were collected from hand-dissected pistils (Le et al., 2010). The fertilized ovules containing zygotes were harvested from siliques 24 h after hand-pollination of emasculated flowers (Le et al., 2010). Finally, after 21–28 days-after-pollination, mature-green embryos and endosperms were hand dissected using fine forceps from seed coat and harvested separately.

In order to track the transmission of epigenetic marks to the next unstressed generation, the control and treated plants were propagated, and tissue samples from 2-week-old seedlings were harvested. Each developmental stage was represented by two biological replicates (15 plants per repeat) that were harvested independently.

### Molecular techniques and methods used in this study

#### Total RNA isolation and purification

Total RNA isolation was done using the Trizol reagent (Invitrogen, Carlsbad, CA) according to the manufacturer's protocol. Total RNA was further purified and concentrated using the RNA Clean-Up and Concentration Kit (Norgen Biotek Corp., Ontario, Canada).

Both, the quality and concentration of every sample were quantified using the NanoDrop 2000C spectrophotometer (Thermo Fisher Scientific Inc.). Agarose gel electrophoresis was performed to verify RNA sample integrity.

#### mRNA deep sequencing

The mRNA libraries were prepared from 16 samples of total RNA in 2 biological replicates for most of the tissues according to the TruSeq RNA sample Prep v2 LS protocol (Illumina, San Diego, CA, U.S.A.). Briefly, mRNA was purified from the total RNA samples using poly-T oligo-attached magnetic beads followed by mRNA fragmentation, first- and second-strand cDNA synthesis. Later, the overhangs resulting from the fragmentation of double-stranded (ds) cDNA were repaired to form blunt ends. A single “A” nucleotide was added to the 3′ ends of the blunt fragments to prevent them from ligating to one another during the adapter ligation reaction. Multiple indexing adapters were ligated to the ends of ds cDNA to prepare them for hybridization onto a flow cell followed by a PCR amplification step. The libraries were quantified using the qPCR technique and analyzed on a Bioanalyzer 2100 (Agilent Technologies) using a DNA specific chip. Subsequently, the libraries were normalized and pooled together followed by flow-cell cluster generation using a cBot fully automated clonal cluster generation system for Illumina sequencing. Single-end multiplexed sequencing was done using the Illumina GAIIx platform with the total of 100 cycles.

#### Deep sequencing of small non-coding RNAs

Small non-coding RNA libraries were generated from the aforementioned tissues using the TruSeq small RNA library construction kit according to the manufacturer's protocol (Illumina, San Diego, CA, U.S.A.). Briefly, the 3′ and 5′ adapters were ligated to small RNAs from the total RNA sample followed by reverse-transcription PCR amplification. PCR was performed with two primers that annealed to the ends of adapters and contained indexes. Subsequently, the libraries with unique indexes were pooled together; the cDNA was gel-purified using a TBE PAGE gel and then concentrated by ethanol. Following a successful library quality control by qPCR, flow cell cluster generation was performed using a cBot. Single-end multiplexed sequencing was done using the Illumina GAIIx platform with the total of 36 cycles.

#### Northern blot analysis of small non-coding RNAs

The confirmation of small RNA sequencing data was performed using a non-radioactive northern blot method as described previously (Kim et al., 2010). Briefly, 3 ug of total RNA was separated on a 15% urea PAGE (National diagnostics, USA) and transferred to a positively charged nylon membrane (Roche). The pre-hybridization was performed with ULTRAhyb Ultrasensitive Hybridization Buffer (Ambion) at 37°C for at least 30 min in the hybridization oven followed by hybridization step with the DIG-labeled probe in ULTRAhyb Ultrasensitive Hybridization Buffer at 37°C overnight with a slow rotation. The DIG-labeled miR168 probe—AB492 (Supplementary Table 9) was synthesized by Eurofins MWG Operon (Huntsville, AL, USA). Subsequently, the membrane was washed and incubated with Anti-Digoxigenin-AP Fab fragments (Roche) followed by the detection using CDP-Star (Roche). The membrane was photographed using the FluorChem HD2 MultiImage™ Light Cabinet (Cell Biosciences Pty *Ltd*, Heidelberg, Australia), and the bands were quantified using the Image J program (NIH, http://rsbweb.nih.gov/ij/).

#### cDNA synthesis and qPCR gene expression analysis

500 ng of total RNA from every tissue in 2 biological replicates was treated with DNase I, purified, converted into cDNA and quantified with qPCR. The real-time quantitative PCR was performed using SsoFast EvaGreen Supermix (Bio-Rad). cDNAs were amplified under the following conditions: (1) 98°C for 2 min for one cycle; 98°C for 5 s, 48°C for 5 s, 65°C to 95°C for 5 s; for 40 cycles; (2) melt-curve analysis −65°C to 95°C for 5 s, with a 0.5°C increment. Primers for the real-time quantitative PCR were designed using the Beacon Designer7 program (Supplementary Table 9). The optimization of the annealing temperature, melt-curve analysis, and gel analysis of amplicons were performed for each set of primers. To evaluate the PCR efficiency, the standard curve was established using a series of cDNA dilutions. Gene expression was confirmed for four differentially expressed genes in the progeny: Bra029235, Bra031065, Bra029719, Bra040903, and Bra032254 (*AtAGO1* homolog). The normalization was done against four *B. rapa* housekeeping genes: *GAPDH*, *TUBULIN*, *EF1α*, and *UBC* (Qi et al., 2010).

The statistical significance between treatment groups was evaluated using the two-tailed paired Student's *t*-test (α = 0.05) and performed using JMP 10.0 software (SAS Institute Inc.).

### The bioinformatic treatment of deep sequencing data

#### mRNA deep sequencing data analysis

Base calling and demultiplexing of transcriptome sequencing reads were performed using the Consensus Assessment of Sequence and Variance (CASAVA) v 1.6 and Novobarcode software (http://www.novocraft.com/). FastQC v 0.10.1 software was used for the preliminary quality check. The reads were mapped to the genome, and *de novo* splice site prediction was performed using TopHat v 2.0.4 beta software (Trapnell et al., 2009). The *de novo* predicted splice-sites obtained were used to perform transcript assembly for each sample separately using Cufflinks v 2.0.2 (Trapnell et al., 2010). The assemblies were merged using the cuffmerge tool in Cufflinks software with the reference file containing *B. rapa* predicted genes (Wang et al., 2011; Trapnell et al., 2012). The aim of this analysis was to compare our *de novo* assembly with the *in silico* predicted transcriptome (Wang et al., 2011).

The merged transcript assembly was used to assess differentially expressed features between treatments with the cuffdiff tool in Cufflinks software (Trapnell et al., 2012). The *q*-value Benjamini-Hochberg method (Benjamini and Hochberg, 1995) below 0.05 was considered as a significant difference of gene expression between treatment groups.

#### Deep sequencing data analysis of small non-coding RNAs

Base calling and demultiplexing of sequencing reads generated by the Illumina GAIIx platform were performed using the CASAVA v 1.8.1. software (http://support.illumina.com/downloads/casava_181.ilmn). Then, the sequencing reads were processed using adapter trimming Cutadapt v 1.1 software (Martin, 2011) with options specified to search for adapter sequences anywhere within the sequencing read and to retain only the sequences that were longer than 17 nucleotides; quality trimming was performed with a Sanger quality cutoff score of 20. Summary statistics and run quality data were collected from the adapter trimmed libraries using FastQC v 0.10.1 software (http://www.bioinformatics.babraham.ac.uk/projects/fastqc/). Samples that passed quality control tests were aligned to the *B. rapa* genome (accession number AENI01000000) (Wang et al., 2011) using Bowtie v 2 2.0.0—beta2 aligner run (Langmead et al., 2009). Bowtie command was: bowtie -v 2 --best -m 50 -p 4 -S <brassica_index> <trimmed_reads> Normalization was performed using DESeq (Anders and Huber, 2010). The reads that could be aligned to the genome were further classified based on feature classes. The alignment was performed in a stepwise fashion. The reads that could be aligned to features of a certain class were counted and excluded from subsequent alignments. In this way, the pool of sequences was gradually depleted. The mapping process continued until the remaining reads could not be assigned to any of the known mapping categories and were labeled as “unclassified.” Both unique reads and reads that matched multiple loci (cutoff ≥ 50 loci for reads mapped to multiple loci) were considered for this analysis.

The alignment to *B. rapa* mature and passenger strand miRNA sequences was performed using conservative and predicted miRNA sequences described before (Yu et al., 2012). A novel miRNA prediction was done by using MiRDeep-P software (Yang and Li, 2011) followed by its alignment to both the predicted *trans*-acting siRNAs (ta-siRNAs) by using the UEA sRNA workbench (Stocks et al., 2012) and non-coding RNA genes, structured *cis*-regulatory elements and self-splicing RNAs from Rfam database, v 10.01 (Burge et al., 2013). The remaining reads were aligned to repeats and predicted genes (*B. rapa* gene database v 1.2). As a result, all small RNAs were sorted into 7 groups: miRNA candidates, gene-aligned small RNAs, conserved miRNAs, Rfam v 10.01, ta-siRNA candidates, transposon-aligned and unclassified small RNAs.

To perform statistical comparisons, the sequence reads were collapsed to unique tags after adapter trimming using a fastx_collapser program from the FASTX-Toolkit (http://hannonlab.cshl.edu/fastx_toolkit/). Raw read counts assigned to unique tags were compared between treatments and tissues. Normalization and statistical tests were performed using DESeq bioconductor package as described in the user's manual (Anders and Huber, 2010). Reads with the sum raw counts ≤ 5 across all libraries that participated in a particular comparison were excluded from the analysis. The cutoff value for significance was *q* < 0.2 (the Benjamini-Hochberg method) (Benjamini and Hochberg, 1995).

The precise mapping of all assorted small RNAs was done using MicroRazerS v 1.0 software with default settings (i.e., the first 16-nt-long ones were matched, no mismatch allowed) (Emde et al., 2010). Only tags that were considered to be significantly changed (*q* < 0.2) were annotated. Due to the repetitive nature of some tag sequences, some single tag sequences had multiple annotations.

#### The prediction of putative gene targets for miRNAs

The psRNATarget software was used with default settings to predict and retrieve gene IDs of putative miRNA gene targets from the *B. rapa* CDS library v 1.1 (Dai and Zhao, 2011). Subsequently, putative gene-target IDs were identified in the differentially expressed gene dataset of the corresponding tissue and annotated using the SWISS-PROT database (Bairoch and Apweiler, 2000).

## Results

### The analysis of gene expression in *B. rapa* parental leaves, inflorescence meristem, pollen, unfertilized ovules, 24-h post-fertilization ovules, embryo, endosperm and leaf tissues of progeny plants after heat shock treatment

The transcriptome libraries were generated and analyzed from the total RNA of somatic and reproductive tissues of control and exposed parental *B. rapa* plants as well as from the untreated progeny (Supplementary Table 1). In the experiment, the genome-matched reads comprised of on average 67.70% of the total reads. Unfortunately, the whole transcriptome sequencing data for *B. rapa* species that could be used for the comparison with our data are not available yet.

**Figure 1:**
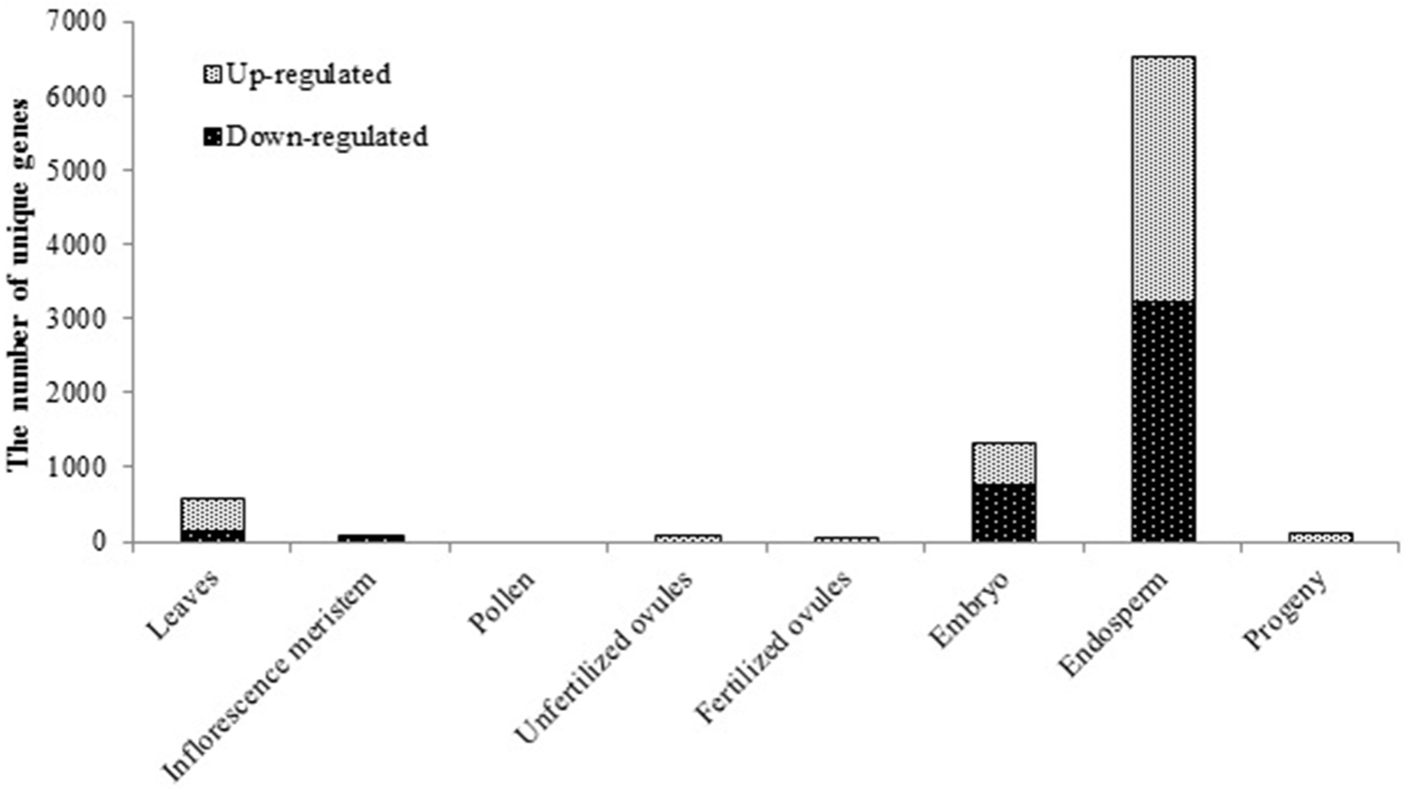
**The effect of exposure to the elevated temperature on gene expression in tissues of parental plants and leaves of untreated progeny plants of *Brassica rapa***. The bars represent the number of unique differentially expressed genes in response to HS in parental tissues and progeny, the Benjamini-Hochberg method, *q* < 0.05.

Unique tissue-specific alterations in mRNA accumulation in heat-shock treated *B. rapa* plants was observed, with a nearly even representation of the number of up- and down-regulated genes (Figure 1). Whereas in the leaves of parental plants that were directly exposed to stress, we detected 562 differentially expressed genes as compared to the untreated controls (the Benjamini-Hochberg method, *q* < 0.05, Figure 1), in the inflorescence meristem derived from the exposed shoot apical meristem, there were only 79 differentially expressed genes as compared to the untreated controls (Figure 1). Both paternal and maternal reproductive tissues responded to HS with little changes in gene expression (3, 78, and 24 differentially expressed genes in pollen, unfertilized ovules and fertilized ovules, respectively, Figure 1).

Enigmatically, the highest oscillations in gene expression were observed in the tissue that was not directly exposed to HS—the endosperm (6365 differentially expressed genes, *q* < 0.05, Figure 1) as compared to control tissue. Such behavior of the endosperm could possibly be attributed to global genome demethylation and a decrease in the expression of silencing-related genes as compared to other tissues, as it was previously reported to occur in the *Arabidopsis* endosperm under normal conditions (Hsieh et al., 2009b; Jullien et al., 2012). Considerable oscillations of gene expression were also observed in the embryo, albeit to a lesser extent than that in the endosperm (1311 differentially expressed genes, *q* < 0.05, Figure 1).

The analysis showed that despite the fact that drastic changes in gene expression were observed in the embryo and endosperm tissues, few changes were passed on to the leaf tissue of progeny when examined at 2 weeks post germination stage (116 differentially expressed genes, *q* < 0.05, Figure 1). This suggests that the reversal of changes in gene expression takes place either during the final steps of seed maturation or later throughout seed germination and plantlet development. At the same time we did not examine the seed tissues from the progeny of stressed plants, hence we cannot rule out the possibility of re-occurrence of similar pattern in embryo and endosperm. Additionally, unfertilized and fertilized ovules collected 24 h post-fertilization also responded to HS treatment with moderate changes in the gene expression profile (Figure 1). Therefore, it may be suggested that alterations in the transcriptional activity of genes occur during stages of cell division/expansion of seed development and proceeds into green embryo and endosperm tissues (Le et al., 2010). Noteworthy, since we did not control the actual fertilization of ovules in the emasculated flowers that were manually pollinated, we were unable to rule out the possibility that fertilized ovule tissue samples could have been contaminated with unfertilized embryo sacs. This could eventually affect the sequencing outcome in the ovule tissue samples collected at 24 h postfertilization. Nevertheless, a comparison of unfertilized and fertilized ovule transcriptomes from control plants revealed 791 significantly differentially expressed genes (*q* < 0.05, the Benjamini-Hochberg method), with 603 being up-regulated in fertilized ovules (data not shown), which suggests that in our experiment, fertilized and unfertilized ovule samples were indeed different. However, it does not completely rule out the presence of unfertilized ovules in fertilized ovule samples.

**Figure 2:**
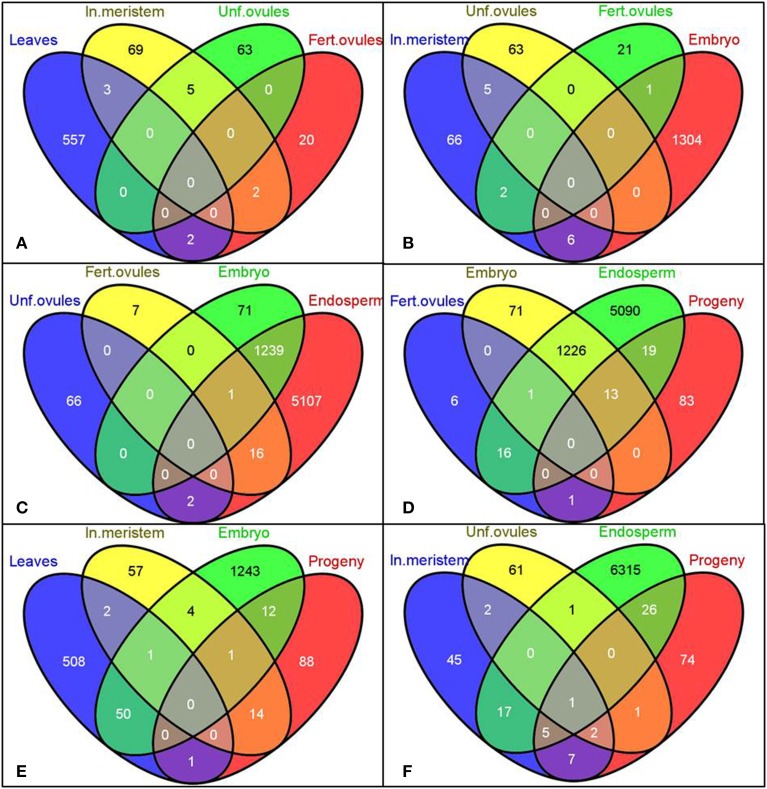
**Venn diagrams representing common genes the expression of which was significantly changed in the selected tissues**. Gene IDs of differentially expressed genes (both up- and down-regulated) were used to create the Venn diagrams using the Venny program (Oliveros). Labels: In. meristem—Inflorescence meristem, Unf. Ovules—Unfertilized ovules, Fert. ovules—Fertilized ovules.

A unique tissue-specific pattern of transcriptome fluctuations following HS stress was further observed in the comparison analysis (Figure 2). The most pronounced overlap of differentially expressed genes was seen between embryo and endosperm (1240 commonly changed genes, Figures 2C,D) and then between embryo and leaves (51 commonly changed genes, Figure 2E). The untreated progeny of plants stressed by HS had the highest overlap of differentially expressed genes with the endosperm (32 commonly changed genes, Figure 2D) followed by the inflorescence meristem (15 commonly changed genes, Figure 2E) and embryo (13 commonly changed genes, Figure 2E). Surprisingly, the embryo and unfertilized and fertilized ovules of stressed parental plants had the lowest number of common differentially expressed genes, which can conceivably be attributed to the overall quiescent response of maternal reproductive organs to stress (Figures 2B,C).

The transcriptome sequencing data were further confirmed in the progeny of stressed and non-stressed plants for four genes with the most significant change in gene expression (log2FC) using the qPCR method (Supplementary Table 2, *q* < 0.05, the Benjamini-Hochberg method).

### Gene ontology annotation of differentially expressed genes in tissues of *B. rapa* plants subjected to heat shock

Differentially expressed genes in all tissues were further grouped into gene ontology categories according to the biological process they participate in Conesa et al. (2005). As expected, the majority of genes in all tissues fall into the gene ontology (GO) category “response to stress” (Supplementary Figures 2, 3), with the only exception of unfertilized ovules (Supplementary Figure 3C) where the GO category “response to stress” was absent. Instead, genes coding for the proteins involved in the overall cell structure development were predominant.

### A small RNA transcriptome analysis of *B. rapa* parental leaves, inflorescence meristem, pollen, unfertilized ovules, 24-h post-fertilization ovules, embryo, endosperm and leaf tissues of progeny plants after heat shock treatment

Illumina small RNA sequencing was performed to compare alterations in smRNA transcriptomes in somatic and reproductive tissues of *B. rapa* plants and in their progeny in response to HS treatment. We hypothesized that it would allow us to reveal possible messengers of transgenerational stress memory inheritance in plants. For smRNA sequencing, we used the same tissue samples as for mRNA sequencing. An average of 4,873,042 reads per library was achieved. Genome-matched reads comprised on average 52.68% of the total reads (Supplementary Table 3), and it was comparable with the previously published report on Chinese cabbage (56.96% of genome-mapped smRNA reads) (Wang et al., 2012). We observed drastic fluctuations in the percentage of genome-mapped sequencing reads: the highest percentage was observed in the inflorescence meristem (79.57% mapped reads on average) and the lowest one—in leaves (25.71% mapped reads on average). Lower mapping rate in some libraries may be attributed to tissue-specific factors, for instance, in tissues containing chlorophyll significant fraction may map to plastid genome. Quick examination of several unaligned reads from progeny of control 1 library, which originates from the leave tissue, showed that they indeed originate from chloroplast genomes.

**Figure 3:**
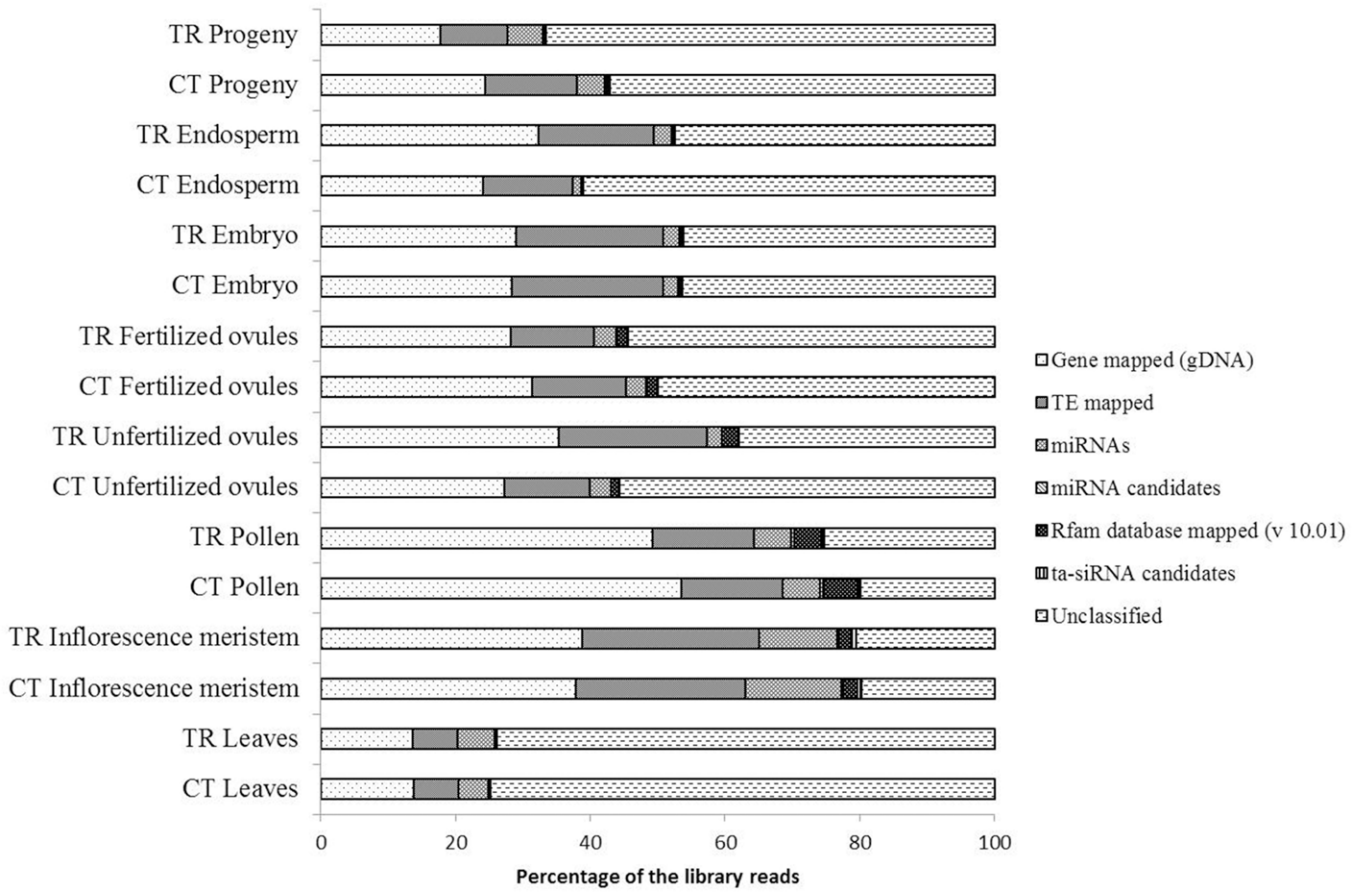
**A general compositional analysis of small RNA libraries sequenced from the total RNA of corresponding tissues**. The length of stacked bars represents the percentage of smRNA fraction occupied by a specific smRNA type in the corresponding library.

A general compositional analysis of smRNA libraries revealed substantial variations in the relative smRNA enrichment among tissues, whereas differences between control and exposed groups within the same tissue were not so pronounced (Figure 3). The overwhelming majority of aligned sequencing reads were mapped to gDNA gene regions (30.29% of library reads on average) followed by those ones mapped to transposons (15.88% of library reads on average) and miRNAs (4.76% of library reads on average). The remaining classified reads comprised of Rfam database (v 10.01) mapped to smRNAs (1.38% of library reads on average), ta-siRNA candidates (0.23% of library reads on average) and miRNA candidates (0.14% of library reads on average). We observed considerable differences in the relative smRNA library composition between parental somatic tissues such as leaves and the inflorescence meristem and between paternal and maternal reproductive tissues.

The most pronounced alterations in the library composition after HS stress were observed in unfertilized ovule tissues with a 9.27 and 8.15% increase in the number of transposon- and gene-mapped smRNA reads as compared to control, respectively (Figure 3). Changes in the endosperm were less pronounced. The progeny of stressed plants had a detectable decrease in the number of smRNA reads mapped to genes (6.66% as compared to control) and to transposons (3.61% as compared to control, Figure 3), which was in accordance with the overall up-regulation of gene expression observed in the progeny of stressed as compared to control plants (Figure 1). The leaf tissues that were directly subjected to stress suffered only minor oscillations in the smRNA library composition as compared to control, albeit both the control and treated smRNA libraries had the highest relative amount of unclassified reads among all tissues that conceivably could contain responsive smRNAs (Figure 3).

### The length distribution of small RNA libraries

Previous reports have shown a conservative pattern of the smRNA length distribution in plants which is compatible with DICER-dependent transcriptome processing (Rajagopalan et al., 2006; Fahlgren et al., 2007; Moxon et al., 2008; Szittya et al., 2008; Song et al., 2010; Chi et al., 2011). We observed the overall bimodal length distribution of smRNA sequencing reads in all tissues (Supplementary Figure 4) with a major peak at 24 nucleotides (40.19% of all reads on average) and a smaller shoulder at 21-nt (18.30% of all reads on average), which was consistent with the published data on *B. rapa* plants (He et al., 2008; Wang et al., 2012). Patterns for the 21- and 24-mers distribution were dissimilar between tissues. In a stark contrast to other tissues, the 21-nt smRNA fraction was predominant in leaves (28.06% on average) followed by 24-nt-long smRNAs (22.20% on average, Supplementary Figure 4A). Also, an equivalent accumulation of 21- and 24-nt-long reads was observed in leaves of 2-week-old progenies (28.42 and 29.55% for 21- and 24-nt-long reads, respectively, Supplementary Figure 4H) that is in agreement with the previous report (He et al., 2008). Whereas the paternal (Supplementary Figure 4C) and maternal (Supplementary Figures 4D,E) reproductive tissues had a different distinguishable pattern of smRNA library length distribution, the length of smRNA reads derived from the inflorescence meristem (Supplementary Figure 4B), embryo (Supplementary Figure 4F) and endosperm (Supplementary Figure 4G) tissues was highly similar. None of examined tissues, except for pollen, responded to HS with detectable fluctuations in the relative smRNA length distribution (Supplementary Figure 4C). The most prominent alterations were observed in the 24-nt-long smRNA fraction (a 1.4 fold increase as compared to control, Student's *t*-test, α = 0.05), which usually corresponds to the smRNA fraction deriving from heterochromatic genomic regions in angiosperms (Axtell, 2013).

The length distribution analysis of sequencing reads mapped to the prominent genomic sequence categories revealed that the majority of gene- (Supplementary Figure 5) and transposon-mapped (Supplementary Figure 6) smRNAs fell mostly into the 24- and 21-nt-long categories in all tissues except for pollen. In male reproductive tissues, a vast amount of low range small RNAs (19-mers and smaller) observed in the total smRNA library were mapped to gene regions in gDNA (17.09 and 16.97% of the relative fraction of 17-nt-long reads in the total smRNA and gene-mapped smRNA libraries, respectively, Supplementary Figure 5C), which suggests that a tissue-specific transcriptome degradation process takes place in pollen. Similar results were previously reported in mature Arabidopsis pollen where 16-nt-long reads were prevailing in the smRNA sequencing library. Unfortunately, the authors did not explain the origin of small size smRNAs in pollen (Grant-Downton et al., 2009b).

smRNAs mapped to transposable elements (TEs) in male tissues were distributed between a major peak of 24-nt long reads (3.96% of the total reads on average) and an unusually broad but minor peak stretching from 17 to 22-nt in length (Supplementary Figure 6C). Previous reports on smRNA metabolism in Arabidopsis pollen (Slotkin et al., 2009) give us a reason to speculate that low range smRNAs can apparently be non-cell autonomous silencing signals generated in the vegetative nucleus to suppress the TE activity in sperm cells.

### Pollen and endosperm of parental plants and leaf tissues of progeny plants demonstrate significant alterations in the small RNAome profile in response to heat shock

**Figure 4:**
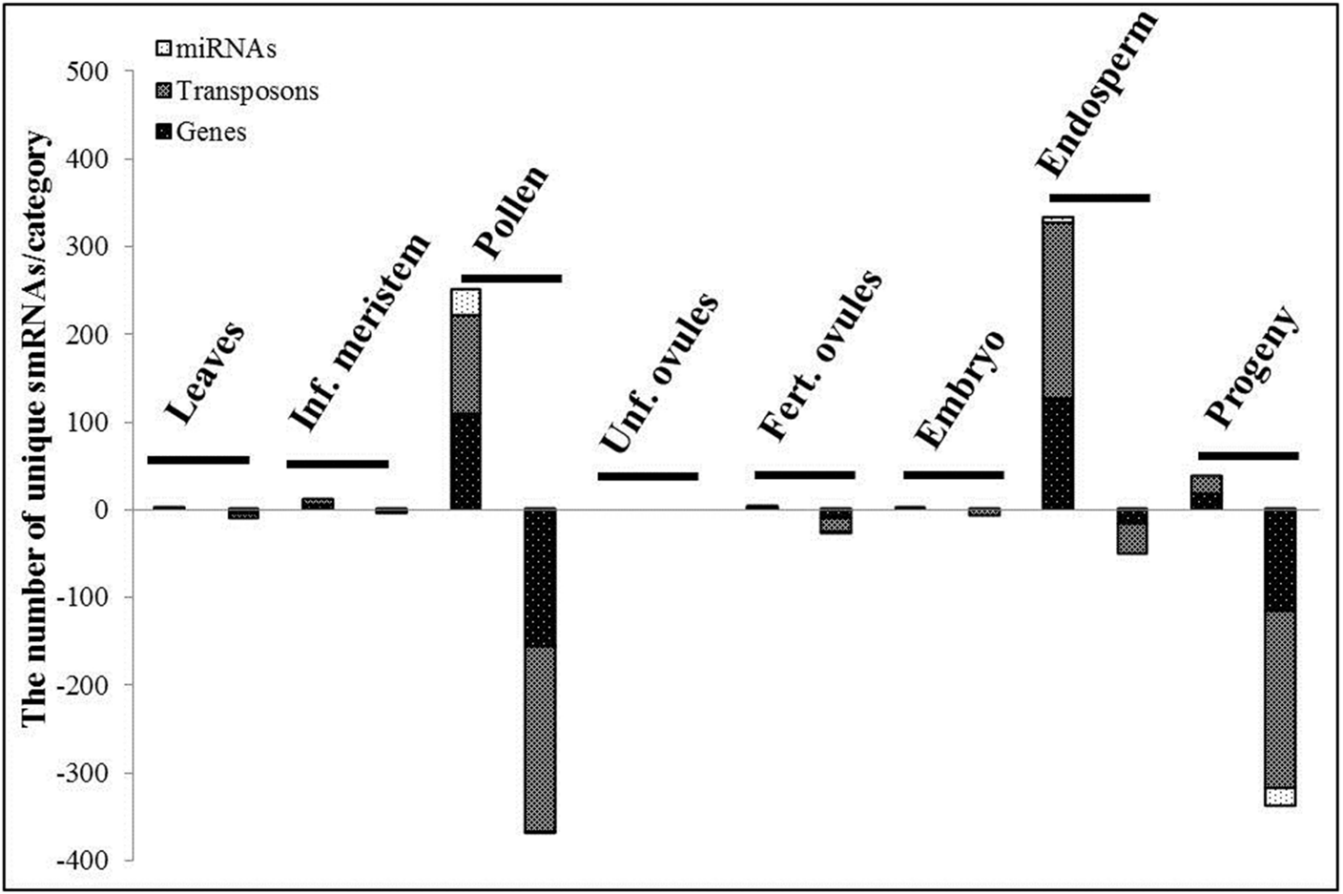
**The differentially expressed small RNAs, TR vs. CT**. The stacked bars represent the number of the unique differentially expressed smRNAs per category in the corresponding tissue. SmRNAs were divided into 3 categories regarding the genome regions they were mapped to: miRNAs, transposon and gene regions. The first and second stacked bars show the number of smRNAs with the positive and negative log2fold change values (TR vs. CT) in the corresponding tissue, respectively (the Benjamini-Hochberg method, *q* < 0.2).

Despite the fact that we did not detect significant fluctuations in the sequence read length distribution in response to HS, considerable changes in the differential expression of smRNAs were observed in all tissues except unfertilized ovules (Figure 4). Surprisingly, the most striking alterations were detected in systemic tissues that were not directly exposed to stress such as pollen (621 differentially expressed smRNAs), the endosperm (385 differentially expressed smRNAs) and more importantly, in leaf tissues of the progeny (376 differentially expressed smRNAs). Minor changes were recorded in leaves (12 differentially expressed smRNAs), the inflorescence meristem (15 differentially expressed smRNAs), fertilized ovules (31 differentially expressed smRNAs) and the embryo (eight differentially expressed smRNAs). Strikingly, none of smRNAs responded to HS in unfertilized ovules (Figure 4, the Benjamini-Hochberg method, *q* < 0.2).

The detailed mapping and analysis of differentially expressed smRNAs showed a lower representation of gene-mapped siRNAs as compared to those ones derived from transposable elements in all tissues (566 and 823 in total for genes and transposons, respectively). Whereas in the endosperm, 86.75% of all differentially expressed smRNAs were up-regulated (Figure 4), in pollen and leaves of the progeny, 59.58 and 89.89% of all differentially expressed smRNAs were down-regulated, respectively. MiRNAs comprised a minor fraction with only 59 of them being differentially expressed *in toto*.

### Differentially expressed siRNAs are mapped to genes that are unique for every tissue

To reveal the commonalities in action of the altered siRNAs mapped to gene regions, we extracted their gene IDs and used them for inter-tissue comparisons. Since TEs are known to get activated in response to environmental perturbations (Grandbastien, 1998), we hypothesized that the levels of TE- and gene-derived siRNAs would be affected by stress exposure, and moreover this response is expected to be tissue-specific.

**Figure 5:**
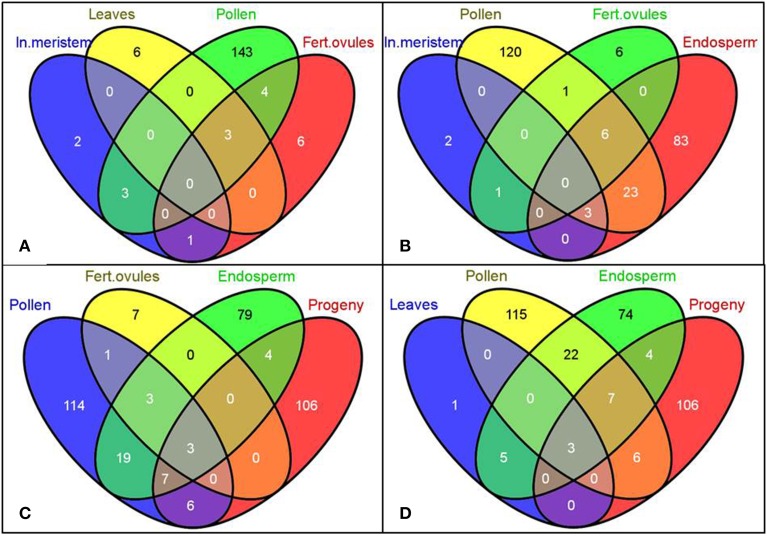
**The Venn diagrams representing common genes mapped to the significantly altered siRNAs among tissues of *B. rapa* plants exposed to heat stress**. Gene IDs of putative gene-mapped siRNA targets were used to create the Venn diagrams using the Venny program (Oliveros)^1^. Labels: In. meristem—Inflorescence meristem, Fert. ovules—Fertilized ovules. **(A–D)** Comparison of common genes mapped to the significantly altered siRNAs among denoted tissues.

In our study, in accordance with our hypothesis, the differentially expressed siRNAs were mostly tissue-specific, with a small number of siRNAs overlapping between pollen and endosperm (32 common genes, Figures 5B–D), pollen and leaf tissues in the progeny (16 common genes, Figures 5C,D), and endosperm and leaf tissues in the progeny (14 common genes, Figures 5C,D). The number of differentially expressed siRNAs in fertilized ovules was very small (11 gene-mapped siRNAs) despite a substantially larger number of them found in pollen (267 gene-mapped siRNAs, Figure 5).

### Expression hot spots of small non-coding RNAs observed in *B. rapa* tissues after heat shock treatment

In our study, we observed hot spots of changes in the expression of smRNAs involved in stress response common for leaves, pollen, fertilized ovules, endosperm and leaves in the progeny (the Benjamini-Hochberg method, *q* < 0.2, Table 1). Despite the fact that the commonly regulated smRNAs were mapped to three different genes (Bra003466, Bra018314, and Bra030669), the sequences of these smRNAs were highly similar because they were produced from the predicted tRNAs which resided in the intron region of the three aforementioned genes. tRNA-derived RNA fragments (tRFs) comprise a novel class of smRNAs discovered recently in plants (Hsieh et al., 2009a; Chen et al., 2011; Hackenberg et al., 2013) and *in silico* predicted to have a regulatory role in gene expression through the miRNA pathway (Loss-Morais et al., 2013). The existence in plants of transgenerationally transmitted, heat-responsive tRFs is a novel finding that adds one more variable to environmentally induced epigenetic responses to stress.

**Table 1:** **The common stress-responsive hot spots mapped to the differentially expressed siRNAs in leaves, pollen, fertilized ovules, endosperm and leaves of the progeny of heat-treated *B. rapa* plants**.

**Tissue**	**Gene ID**	**siRNA origin**	**Average siRNA expression log2 FC, TR vs. CT**
Leaves	Bra003466	Intron/tRNA-Gly	−1.57
	Bra018314	Intron/tRNA-Ala	−1.71
	Bra030669	Intron/tRNA-Gly	−1.71
Pollen	Bra003466	Intron/tRNA-Gly	2.40
	Bra018314	Intron/tRNA-Ala	−0.18
	Bra030669	Intron/tRNA-Gly	2.40
Fertilized ovules	Bra003466	Intron/tRNA-Gly	−6.46
	Bra018314	Intron/tRNA-Ala	−6.46
	Bra030669	Intron/tRNA-Gly	−6.46
Endosperm	Bra003466	Intron/tRNA-Gly	8.18
	Bra018314	Intron/tRNA-Ala	8.31
	Bra030669	Intron/tRNA-Gly	8.18
Progeny	Bra003466	Intron/tRNA-Gly	6.95
	Bra018314	Intron/tRNA-Ala	2.59
	Bra030669	Intron/tRNA-Gly	4.61
SWISS-PROT annotation Bra003466	BECN1_ARATH Beclin-1-like protein OS=*Arabidopsis thaliana* GN=At3g61710 PE=2 SV=2		
SWISS-PROT annotation Bra018314	N/A		
SWISS-PROT annotation Bra030669	CIA2_ARATH Protein CHLOROPLAST IMPORT APPARATUS 2 OS=*Arabidopsis thaliana* GN=CIA2 PE=2 SV=1		

The common differentially expressed smRNAs (the Benjamini-Hochberg method, q < 0.2) were mapped to the genome, and positional coordinates on the B. rapa chromosomes were extracted. Subsequently, using the coordinates of the region, genes and tRNAs were allocated in the BRAD—Brassica Genome Browser v 1.2 (http://brassicadb.org/cgi-bin/gbrowse/Brassica/).

### The functional annotation of gene-mapped siRNAs

To examine common biological pathways that are presumably affected by differentially expressed smRNAs mapped to genes, their putative targets were further annotated and classified according to the biological process they were involved in. To do this, we included only the data sets of the three tissues that demonstrated the maximum smRNAome disequilibrium after HS—pollen, endosperm and leaf tissues of the progeny. Due to the repetitive nature of the *B. rapa* genome, a number of differentially expressed sequencing reads were mapped to multiple genomic loci. Also, a vast majority of siRNAs originated from the intron sequences apparently do not have an ability to regulate gene expression at the posttranscriptional level, excluding the alternative transcripts that rarely occur in plants.

**Figure 6:**
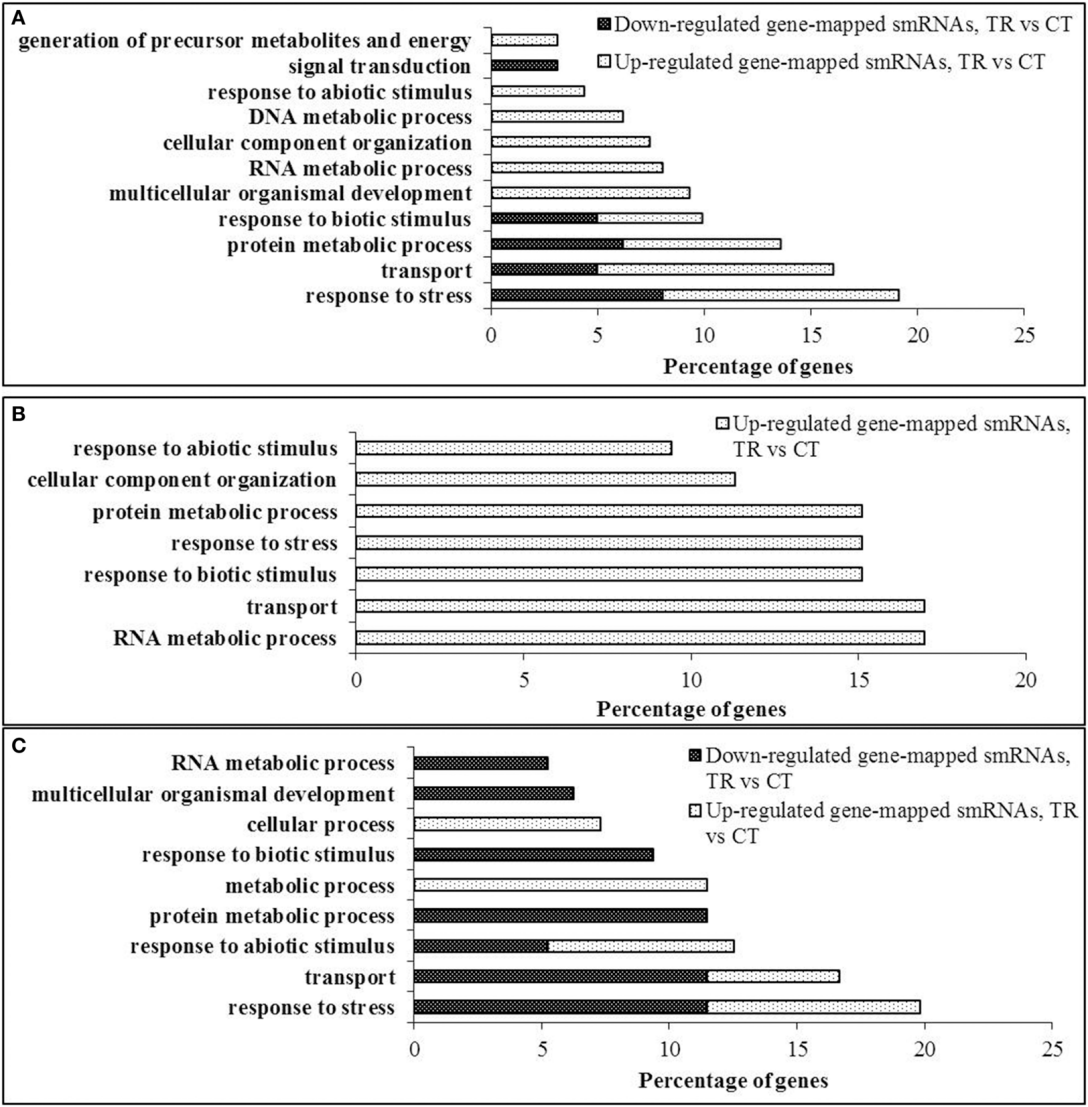
**Gene ontology annotation of putative siRNA targets. (A)** Pollen, **(B)** Endosperm, **(C)** Progeny. Coding sequences of putative targets of the differentially expressed gene-mapped siRNAs were extracted from the *B. rapa* transcriptome database v 1.2 and loaded as a FASTA file into the Blast2GO v 2.6.2 software for the NCBI BLAST similarity search using a blastx option (Conesa et al., 2005). Further, the recovered ontologies were annotated and grouped into gene ontology categories using default settings. Gene ontology nodes were combined into the most prominent categories using a GO-slim-TAIR tool and represented as bar graphs.

Overall, whereas the genes involved in “response to stress” were the predominant putative targets in pollen and leaves of the progeny tissues (19.13 and 19.78%, respectively, Figures 6A,C); in endosperm, smRNAs mapped to the genes involved in “RNA metabolic process” and “transport” were the most enriched (Figure 6B).

### Common differentially expressed miRNAs in examined tissues

The analysis of siRNA fractions of sequencing reads was followed by a detailed examination of those reads mapped to conservative plant micro RNA (miRNA) sequences described in the recent paper (Yu et al., 2012) and those ones that were *de novo* predicted (Yang and Li, 2011). The unique sequences mapped to miRNAs constituted a minor fraction of differentially expressed smRNAs that responded to HS regardless of the tissue sample. The largest number of altered mature miRNA sequences were observed in pollen (31 miRNAs, 4.99% of all altered smRNAs, Supplementary Table 4), in the endosperm (6 miRNAs, 1.56% of all altered smRNAs, Supplementary Table 5) and in the progeny (20 miRNAs, 5.32% of all altered smRNAs, Supplementary Table 6).

Only one miRNA was differentially expressed in the inflorescence meristem and fertilized ovules as compared to controls (Supplementary Table 7), and none of altered miRNAs was observed in the unfertilized ovules, embryo and leaves of heat stressed *B. rapa* plants (the Benjamini-Hochberg method, *q* < 0.2).

Our analysis allowed us to predict one novel miRNA in the pool of differentially expressed smRNAs in pollen (miR22711, Supplementary Table 4) and two novel miRNAs in the progeny (miR31241, miR315691, Supplementary Table 6), suggesting that the potential for the sequencing-based discovery of novel miRNAs in *B. rapa* plants is not exhausted.

**Figure 7:**
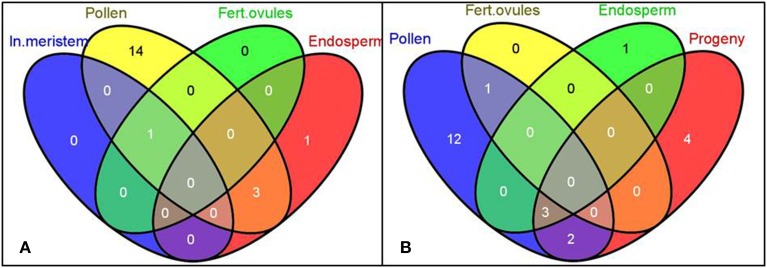
**The Venn diagrams representing the common microRNA gene families of differentially expressed mature miRNAs among tissues in heat-stressed *B. rapa* plants as compared to controls**. The differentially expressed smRNAs mapped to conservative miRNAs (TR vs. CT, *q* < 0.2, the Benjamini-Hochberg method) were grouped into microRNA gene families and used for the generation of Venn diagrams using the Venny software. Labels: In. meristem—Inflorescence meristem, Fert. ovules—Fertilized ovules. **(A,B)** Comparison of common microRNA gene families of differentially expressed mature miRNAs among denoted tissues.

MicroRNAs are known to play an important role in plant stress response since they act as global regulators of gene expression (Kruszka et al., 2012). More importantly, recently miRNAs have been implicated in a non-cell autonomous mode of action (Carlsbecker et al., 2010; Marin et al., 2010). This raises a question whether miRNAs generated in the directly exposed tissues can be mobilized to distinct systemic organs, such as reproductive tissues, and modulate the inheritance of transgenerational stress memory. Hence, we performed the commonality analysis of miRNAs between sequencing libraries of parental plants and untreated progeny. We observed a unique pattern of tissue response to stress (Figure 7A), with only a few overlapping differentially expressed mature miRNAs among pollen, endosperm and leaf tissues of the progeny belonging to three microRNA gene families: bra-miR167, bra-miR390, and bra-miR168 (Figure 7B, Table 2). The members of miR167 and miR390 gene families have been implemented in the regulation of auxin response factors (ARFs) in Arabidopsis which are transcription factors that bind to auxin response elements in the promoters of early auxin response genes (Tiwari et al., 2003; Mallory et al., 2005; Montgomery et al., 2008). The discovery of differential expression of miR168 that regulates *ARGONAUTE 1* (*AGO1*) level in Arabidopsis was the most intriguing (Vaucheret et al., 2004). *AGO1* has been shown to be vital for plant development due to its unique and essential role in microRNA metabolism. Hence, the discovery of altered expression of miR168 in parental *B. rapa* plants exposed to heat stress and in the untreated progeny makes it tempting to speculate about its role in transgenerational epigenetic inheritance of stress memory. Consequently, the members of the miR168 microRNA gene family were selected for further analysis, and their expression was related to the *AGO1* transcript level in both parental plants exposed to stress and the untreated progeny.

**Table 2:** **The common significantly altered microRNA gene families in the pollen, endosperm and leaves of progeny of heat-stressed plants**.

**The miRNA gene family**	**miRNA**	**Tissue**	**Log2 fold change, TR vs. CT**	***q*-value**
bra-miR167	bra-miR167d	Pollen	1.19	5.00E-02
		Endosperm	4.37	5.00E-02
		Progeny	−3.29	1.30E-01
bra-miR390	bra-miR390a-1	Pollen	2.32	2.00E-02
		Endosperm	3.78	1.90E-01
		Progeny	−2.69	4.00E-04
bra-miR168	bra-miR168a-1	Pollen	1.18	4.00E-02
	bra-miR168a-3	Endosperm	6.48	5.00E-02
	bra-miR168a-1	Progeny	−1.57	1.90E-01

The differentially expressed smRNAs mapped to conservative mature miRNAs (TR vs. CT, q < 0.2, the Benjamini-Hochberg method).

### miR168 is a putative messenger of transgenerational epigenetic inheritance

Simply detecting miRNAs in stressed parental tissues and untreated progeny does not provide evidence that they are functional as smRNAs and can guide targeting RNA and DNA substrates (Grant-Downton and Rodriguez-Enriquez, 2012). Hence, we proceeded with the examination of putative targets of differentially expressed miRNAs in parental tissues and the progeny. Since the endosperm was the most responsive tissue to stress at the level of gene and smRNA expression (see Figures 1, 4), it conceivably led to the highest number of smRNA/mRNA target pairs identified. Six smRNA/mRNA target pairs were identified in the endosperm of stressed plants (Table 3), with the most intriguing of them being bra-miR168/*AGO1* (Max identity = 95%, *E*-value = 2E-99 for *atAGO1* and *braAGO1* BLAST search). Curiously, the expression of *braAGO1* was also significantly altered in the embryo of stressed plants as compared to controls (Table 4, the Benjamini-Hochberg method). Overall, we observed a bimodal tissue-specific trend in the expression of *braAGO1* after HS stress in parental plants. Whereas an insignificant down-regulation was observed in leaves, unfertilized and fertilized ovules, the up-regulation was detected in the inflorescence meristem and pollen (Table 4, the Benjamini-Hochberg method). The absence of negative correlation for significantly overexpressed miR168 and *braAGO1*, observed in our study in pollen, is in contrast with the previous report showing that the regulation of *AGO1* expression by miR168 is active in mature Arabidopsis pollen grains (Grant-Downton et al., 2009a).

**Table 3:** **Changes in the expression of miRNAs and their putative targets in response to heat stress in the endosperm of *B. rapa* plants**.

**miRNA**	**miRNA log2FC, TR vs. CT**	**Gene ID of the putative target**	**Gene log2FC, TR vs. CT**	**The predicted miRNA's mode of action**	**SWISS-PROT annotation**
bra-miR167	4.37	Bra015704	0.86	Translation inhibition	TM1L2_XENLA TOM1-like protein 2 OS=*Xenopus laevis* GN=tom1l2 PE=2 SV=1
bra-miR167	4.37	Bra002277	−1.25	mRNA cleavage	TDRD3_CHICK Tudor domain-containing protein 3 OS=Gallus gallus GN=TDRD3 PE=2 SV=1
bra-miR167^*^	5.29	Bra025064	5.84	mRNA cleavage	GDL82_ARATH GDSL esterase/lipase At5g45670 OS=*Arabidopsis thaliana* GN=At5g45670 PE=2 SV=1
bra-miR167^*^	5.29	Bra005019	2.28	Translation inhibition	RCA_ARATH Ribulose bisphosphate carboxylase/oxygenase activase, chloroplastic OS=Arabidopsis
bra-miR168	6.48	Bra032254	−1.56	mRNA cleavage	AGO1_ARATH Protein argonaute OS=*Arabidopsis thaliana* GN=AGO1 PE=1 SV=1
bra-miR171a-1	14.89	Bra039431	0.79	Translation inhibition	PRS6A_BRACM 26S protease regulatory subunit 6A homolog OS=Brassica campestris GN=TBP1 PE=2 SV=1

Gene target prediction for miRNAs was done using the psRNATarget program for the Brassica rapa CDS library v 1.1 (Dai and Zhao, 2011). Log2FC—log2fold change, TR vs. CT. The statistical significance was calculated using the Benjamini-Hochberg method, q < 0.2.

* denotes a complementary strand of the corresponding mature miRNA.

**Table 4:** **The validation of bra-miR168a-1 and *braAGO1* expression in tissues of heat-shock-stressed *B. rapa* parental plants and untreated progeny**.

**Tissue**	**log2FC TR vs. CT, bra-miR168a-1 expression, sequencing**	***q*-value, *sequencing***	**Normalized log2FC TR vs. CT, bra-miR168a-1 expression, Northern Blot**	**log2FC TR vs. CT *braAGO1* expression, sequencing**	***q*-value, *sequencing***	**log2FC TR vs. CT *braAGO1* expression, qPCR**	***p*-value, TR vs. CT, qPCR**
Leaves	0.58	1	3.15	−0.08	1	0.74^*^	2.13E-02
Inflorescence meristem	0.05	1	−0.07	0.41	1	0.69^*^	6.00E-04
Pollen	1.19^*^	0.04	0.36	0.64	1	1.08^*^	2.01E-04
Unfertilized ovules	−0.54	1	−0.21	−0.08	1	−0.81^*^	5.04E-03
Fertilized ovules	−0.62	1	0.64	−0.04	1	0.70^*^	1.26E-04
Embryo	0.61	1	2.50	−0.77^*^	1.29E-02	−0.60^*^	4.12E-03
Endosperm	3.42^*^	0.41	1.16	−1.56^*^	1.36E-04	−0.81^*^	1.23E-03
Progeny	−0.58^*^	0.93	−1.12	0.06	1	0.57^*^	2.03E-02
Correlation, *braAGO1* expression vs. bra-miR168a-1, sequencing data	Pearson's *r* = −0.66						

The asterisks denote a significant difference in the expression as compared to controls (the Benjamini-Hochberg method, q < 0.2 and the Student's t-test, P < 0.05 for sequencing and qPCR data, respectively). Log2FC—estimated log2 value of fold change for treated plants vs. controls.

The expression of *braAGO1* was similar in the progeny of treated and untreated plants, albeit a significant down-regulation of miR168 was detected (Table 4, the Benjamini-Hochberg method). An inverse correlation for miR168 expression and *atAGO1* transcript levels under stress conditions is not always obvious in Arabidopsis, because both *atAGO1* and miR168 promoters are activated under abiotic stress conditions, suggesting that an increase in the miR168 level is essential for retaining a stable *AGO1* transcript level during stress response (Li et al., 2012). We also observed a moderate negative correlation for bra-miR168a-1 and *braAGO1* expression in all tissues of stressed parental plants and untreated progeny of *B. rapa* (Pearson's *r* = −0.66, Table 4).

Unexpectedly, in the most of differentially expressed miRNAs, we did not find an obvious negative correlation with their putative gene targets in the endosperm (Table 3). On the contrary, both miRNAs and their target genes demonstrated an increase in the expression following HS. It is possible that these miRNAs function at the level of translational inhibition rather than the level of mRNA cleavage, although the latter mechanism is believed to prevail in plants (Axtell, 2013).

A detailed analysis of mature miRNA sequences belonging to the miR168 gene family revealed a common 20-nt-long core sequence between 3 miRNAs with only a variable 3′-terminal nucleotide (Supplementary Table 8). The validation of miR168 expression was performed using smRNA Northern blot analysis with a probe designed to recognize the consensus sequence of the three mature miRNAs (Supplementary Figure 7).

## Discussion

### The embryo and endosperm demonstrate the most pronounced oscillations in the trancriptome profile after heat shock stress

A tissue-specific perturbation of gene expression in response to stress exposure has been previously shown in a number of plant species including *Arabidopsis* (Prandl et al., 1995; Nylander et al., 2001; Iyer-Pascuzzi et al., 2011), wine grape *Vitis vinifera* (Tillett et al., 2011), diploid cotton *Gossypium arboretum* (Zhang et al., 2013), *Nicotiana plumbaginifolia* (Castresana et al., 1990) and *Brassica napus* (Dong et al., 2004). In the current study, we observed tissue-dependent fluctuations of the transcriptome in response to stress in *B. rapa* plants (Figures 1, 2). Additionally, we demonstrated a comprehensive profile of gene expression following heat shock in somatic and reproductive parental tissues and in the untreated progeny of exposed plants. More importantly, we found that the highest oscillations of gene expression were observed not in parental tissues that were directly exposed to stress (such as leaves) but in the developmentally distant untreated seeds, suggesting the existence of a mitotically and meiotically transmitted signal of plant stress response (Figure 1). A handful of messengers have been implicated in heat stress response (HSR) in plants that include reactive oxygen species (ROS) (Larkindale and Huang, 2004; Larkindale et al., 2005a), Ca^2+^ cations (Liu et al., 2005), and phytohormones such as abscisic acid (ABA), salicylic acid (SA) and ethylene (Larkindale and Huang, 2004; Larkindale et al., 2005a,b). At present, only ROS was shown to mediate systemic signaling in response to heat stress (Miller et al., 2009), albeit the other mediators are also likely contribute to long-distance signaling in plants (Heil and Ton, 2008; Jung et al., 2009). Regardless of the signal's nature, in our experiments, their action resulted in the priming of an array of genes in somatic and reproductive tissues of stressed plants that enigmatically culminated in a burst of transcription changes in the embryo and endosperm (Figure 1). Alternatively, less pronounced HSR of gene expression observed in the inflorescence meristem and reproductive tissues of stressed *B. rapa* plants indicates a more stringent regulation of gene expression in these tissues as compared to the embryo and endosperm (Figure 1).

Unfortunately, reports indicating transcriptome changes in reproductive tissues of plants in response to stress are scarce. A single study conducted on mature pollen treated with 0°C for 72 h reported insignificant oscillations in the transcriptome profile of pollen as compared to vegetative leaf tissues (Lee and Lee, 2003). This is consistent with our data demonstrating only three genes to be differentially expressed following HS treatment in pollen (Figure 1). On the other hand, the lack of substantial oscillations in the transcriptome profile in exposed ovules as compared to controls can be simply due to the abortion of severely affected ovules to facilitate shunting of resources from reproductive activities into metabolic reactions that increase stress tolerance (Sun et al., 2004; Young et al., 2004; Hedhly, 2011). The remaining ovules that survived apparently acquired an epigenetic signal that was transmitted to the embryo and endosperm. Curiously, 1240 of differentially expressed genes were common in the stressed embryo and endosperm, (94.58 and 19.48%, respectively), suggesting that the additional maternal genome contributes to substantial fluctuations in the endosperm transcriptome (Figures 2C,D). This finding is in agreement with the previous report demonstrating that the maternal genome in the Arabidopsis endosperm is substantially less methylated than the paternal genome in the CpG context (Ibarra et al., 2012).

Numerous examples of transgenerational inheritance in angiosperms undoubtedly suggest the existence of certain messengers in plants (Boyko et al., 2010; Bilichak et al., 2012; Luna et al., 2012; Rasmann et al., 2012; Slaughter et al., 2012). As such, they presumably act to prime the stress-specific genes for providing faster and more pronounced changes in transcription if akin exposure is encountered by offspring of stressed plants (Kathiria et al., 2010; Luna et al., 2012). Consistent with this notion, we detected a higher enrichment of stress-related genes in the fraction of differentially expressed genes in the progeny of stressed plants as compared to the parental tissues, which argues against a stochastic nature of epigenome variability (54.29% of stress-related genes out of the total number of differentially expressed genes, Supplementary Figure 3C).

### Pollen exhibits tissue-dependent smRNAome fluctuations in response to heat shock

In numerous flowering plants examined to date, 24-nt-long heterochromatic siRNAs (hc-siRNA) comprise the overwhelming majority of smRNA transcriptome (Nobuta et al., 2008; Wu et al., 2010; Korbes et al., 2012; Axtell, 2013). In our study, a vast amount of smRNAs were in the 24-nt-long fraction followed by the 21-nt-long fraction of reads in all libraries except leaves (Supplementary Figure 4).

Mapping of smRNAs to transposable elements and genic regions has confirmed the previous findings that the 24-nt smRNAs encompass a major fraction of siRNAs for all tissues except pollen (Supplementary Figures 5, 6). Pollen-derived sequencing reads demonstrated a singularity of smRNA length distribution mapped to the gene and transposon regions as compared to other *B. rapa* tissues. Gene-mapped smRNAs demonstrated the accumulation of sequencing reads with a length less than 19-nt (Supplementary Figure 5C), conceivably the products of mRNA degradation or as-yet-undiscovered pollen-specific regulatory RNAs. In Arabidopsis pollen, the 21-22-nt-long smRNAs are predominantly generated in the vegetative nucleus which is sacrificed by allowing rampant transposon expression concomitant with global DNA demethylation (Slotkin et al., 2009; Calarco et al., 2012). Subsequently, these 21-22-nt siRNAs guide RNA-dependent DNA methylation at non-symmetrical CpHpH sequences in sperm cells in order to reinforce the silencing of transposons. Neither unfertilized nor fertilized ovules responded with significant fluctuations in the smRNA length distribution, albeit the smRNA pathways were shown to be functional in Arabidopsis egg cells (Supplementary Figures 4D,E) (Wuest et al., 2010). Curiously, the fertilization of the embryo sac resulted in slight perturbations in the smRNA profile with a vast majority of repeat-derived sequencing reads (19 out of 27 unique sequencing reads) as compared to controls and unfertilized ovules (Figure 3). Whereas a comparison of unique altered sequencing reads between pollen and fertilized ovules returned only one common siRNA, seven out of 14 genes were found to be a common source of siRNAs in pollen and fertilized ovules. This finding partially confirms a previous report that the vegetative nucleus which does not contribute genetic material to the progeny is the primary source of smRNAs in pollen (Calarco et al., 2012). Also, it may indicate a transcriptionally quiescent response of the embryo sac to stress in plants that can be a prerequisite for the maintenance of genome stability in the harsh environmental conditions.

### miR168 is a putative messenger of transgenerational stress memory inheritance in *B. rapa* plants

Whereas siRNAs, with a few exceptions (Dunoyer et al., 2010; Mccue et al., 2012), are known to suppress predominantly TE activity in the genome, miRNAs are well-characterized regulatory elements of gene expression in plants and animals (Axtell, 2013).

The Arabidopsis genome encodes 10 AGO proteins, most of which demonstrate a clear bias toward a specific class of smRNAs depending on the size and 5′-terminal nucleotide composition (Vaucheret, 2008). One of the AGO proteins—AGO1—plays a principal role in both the siRNA- and miRNA-guided modulation of gene activity (Bohmert et al., 1998; Morel et al., 2002; Kidner and Martienssen, 2004). As a result of the global importance of AGO1 in plant homeostasis and development, its expression is firmly modulated by negative feedback loops involving miR168 and AGO1-derived siRNAs (Vaucheret et al., 2004, 2006; Mallory and Vaucheret, 2009; Mallory et al., 2009). The former regulation pathway is of a particular interest since miR168 expression is altered by numerous environmental perturbations in a number of plant species (Li et al., 2008, 2012; Ding et al., 2009; Jia et al., 2009, 2010; Zhou et al., 2010; Sunkar et al., 2012). Additionally, the involvement of *AGO1* and the microRNA pathway in the adaptation to repeated heat stress has been demonstrated at the physiological and molecular level in Arabidopsis (Stief et al., 2014). In our study, we observed a differential expression of bra-miR168 following HS in the parental tissues that was negatively correlated with *braAGO1* transcript levels in the corresponding tissues (Table 4). Although it still remains to be validated whether *braAGO1* maintains the same functions as the Arabidopsis homolog, it is an interesting finding that conceivably suggests that miR168 and AGO1 are possible bandmasters of transgenerational stress memory inheritance in plants. Consistent with this notion, we observed an inverse Pearson's correlation for the expression of *braAGO1* gene and alterations in the transcriptome profile of the corresponding tissues in the parental exposed plants and untreated progeny (*r* = −0.89, for the total number of differentially expressed genes and *braAGO1* log2fold changes, TR vs. CT in the corresponding tissues). At the same time, we did not observe a Pearson's correlation comparable with the *braAGO1* for the other epigenetic-related genes: putative *DNA-DIRECTED RNA POLYMERASE E*, *r* = 0.65; putative methyltransferase *CMT2*, *r* = 0.63; and putative lysine-specific demethylase *JMJ14*, *r* = −0.67.

Consistent with the previous study in Arabidopsis (Zhong et al., 2013) the overwhelming majority of differentially expressed smRNAs were down-regulated in the progeny of stressed plants as compared to controls (338 out of 376, Figure 4), which was concomitant with a slight up-regulation of gene expression (Figure 1). Taking into consideration that smRNAs act strictly by the down-regulation of gene expression, we can speculate that their progressive depletion provides a capacity for an organism to up-regulate rapidly the required gene expression under stress conditions without the ultimate smRNA-mediated transcript degradation. Furthermore, the removal of such post-transcriptional restraints along with locus-specific demethylation can be the cause of transgenerational priming of stress-responsive genes previously described in offspring of stressed plants (Luna et al., 2012).

Overall, by using a massive parallel sequencing technology, we provide evidence of transgenerational stress memory inheritance both at the transcriptome and smRNAome levels in plants. More importantly, we also suggest that miR168 is a possible messenger that mediates meiotic epigenetic inheritance in plants. Further experiments on transgenerational stresses involving the Arabidopsis hypomorphic *ago1* mutants will shade a new light on its contribution to epigenetic inheritance in plants.

### Conflict of interest statement

The authors declare that the research was conducted in the absence of any commercial or financial relationships that could be construed as a potential conflict of interest.

## Footnotes

^1^Oliveros, J. VENNY. An interactive tool for comparing lists with Venn diagrams.
