# Supplementary material for: The elucidation of stress memory inheritance in Brassica rapa plants
Source: Front Plant Sci. 2015 Jan 21;6:5. doi: 10.3389/fpls.2015.00005 (PMC4300914; doi:10.3389/fpls.2015.00005)
Supplement: Supplementary file 1 [file DataSheet1.DOCX]

1. **Supplementary information**

**Supplementary table 1. The characteristics of transcript reads of control and heat shock treated tissues of *B. rapa* plants**

| **Library ID** | **Description** | **The total number of library reads** | **Percentage of reads mapped to gDNA** |
| --- | --- | --- | --- |
| CT Leaves | Leaf samples from untreated plants | 8,913,668 | 64.13 |
| TR Leaves | Leaf samples from exposed plants | 8,111,316 | 64.48 |
| CT Inflorescence meristem | Inflorescence meristem of untreated plants | 13,992,893 | 65.29 |
| TR Inflorescence meristem | Inflorescence meristem of exposed plants | 21,031,116 | 78.38 |
| CT Pollen | Mature pollen from untreated plants | 6,128,711 | 66.54 |
| TR Pollen | Mature pollen from exposed plants | 14,386,477 | 81.49 |
| CT Unfertilized ovules | Ovules from emasculated flowers of untreated plants | 10,112,352 | 62.28 |
| TR Unfertilized ovules | Ovules from emasculated flowers of exposed plants | 15,684,059 | 74.35 |
| CT Fertilized ovules | 24-hour seeds from emasculated pollinated flowers of untreated plants | 16,766,263 | 73.99 |
| TR Fertilized ovules | 24-hour seeds from emasculated pollinated flowers of exposed plants | 10,461,971 | 67.54 |
| CT Embryo | Mature green embryo dissected from seeds of untreated plants | 9,261,796 | 67.67 |
| TR Embryo | Mature green embryo dissected from seeds of exposed plants | 10,167,508 | 67.58 |
| CT Endosperm | Mature green endosperm dissected from seeds of untreated plants | 9,867,388 | 61.58 |
| TR Endosperm | Mature green endosperm dissected from seeds of exposed plants | 10,393,140 | 63.92 |
| CT Progeny | Leaf samples from the progeny of untreated plants | 12,352,719 | 61.89 |
| TR Progeny | Leaf samples from the progeny of exposed plants | 9,942,780 | 62.11 |
| Average |  | 11,723,384 | 67.70 |

**Supplementary table 2. Validation of transcriptome sequencing data in the leaves of progeny of control and stressed plants of *B. rapa* using qPCR**

Genes which demonstrated the most prominent differential expression between control and treatment groups in the leaves of the progenies of stressed plants were selected for validation using qPCR. The average of four reactions (two dilutions per each of two cDNA preparations streaming from two independent tissue samples) was obtained, and the normalized expression ratio was calculated using 2^-ΔΔCT^ method. The statistical significance of sequencing reads and qPCR expression analysis was assessed using the Benjamini-Hochberg method (q<0.05) and Student’s t-test (p<0.05), respectively.

| **Gene ID** | **log2 Fold change TR vs CT, sequencing** | **q-value, sequencing** | **log2 Fold change TR vs CT, qPCR** | **p-value, qPCR** | **Significant** | **SWISS-PROT annotation** |
| --- | --- | --- | --- | --- | --- | --- |
| Bra029235 | 5.98 | 1.50E-04 | 5.37 | 5.03E-04 | Yes | BAT42_CAEEL BTB and MATH domain-containing protein 42 OS=*Caenorhabditis elegans* |
| Bra031065 | 5.96 | 1.92E-03 | 3.79 | 9.04E-03 | Yes | TI10A_ARATH Protein TIFY 10A OS=*Arabidopsis thaliana* |
| Bra029719 | -4.65 | 3.43E-02 | -3.71 | 2.25E-03 | Yes | NLTP6_ARATH Non-specific lipid-transfer protein 6 OS=*Arabidopsis thaliana* |
| Bra040903 | -3.53 | 4.37E-07 | -3.24 | 1.69E-02 | Yes | UBP13_ARATH Ubiquitin carboxyl-terminal hydrolase 13 OS=*Arabidopsis thaliana* |

**Supplementary table 3. Small RNA sequencing libraries generated from the total RNA of control and heat shock treated tissues of *B. rapa* plants**

| **Library ID** | **Description** | **The total number of library reads** | **Percentage of reads aligned to gDNA elements** |
| --- | --- | --- | --- |
| CT Leaves | Leaf samples from untreated plants | 3,380,794 | 25.22 |
| TR Leaves | Leaf samples from exposed plants | 3,483,667 | 26.20 |
| CT Inflorescence meristem | The inflorescence meristem of untreated plants | 9,127,684 | 80.29 |
| TR Inflorescence meristem | The inflorescence meristem of exposed plants | 4,831,492 | 78.84 |
| CT Pollen | Mature pollen from untreated plants | 5,465,212 | 80.18 |
| TR Pollen | Mature pollen from exposed plants | 5,221,604 | 74.63 |
| CT Unfertilized ovules | Ovules from emasculated flowers of untreated plants | 4,474,129 | 44.49 |
| TR Unfertilized ovules | Ovules from emasculated flowers of exposed plants | 2,462,572 | 69.45 |
| CT Fertilized ovules | 24-hour seeds from emasculated pollinated flowers of untreated plants | 14,151,236 | 49.94 |
| TR Fertilized ovules | 24-hour seeds from emasculated pollinated flowers of exposed plants | 2,660,009 | 44.44 |
| CT Embryo | Mature green embryo dissected from the seeds of untreated plants | 3,827,441 | 53.64 |
| TR Embryo | Mature green embryo dissected from the seeds of exposed plants | 4,927,259 | 53.87 |
| CT Endosperm | Mature green endosperm dissected from seeds of untreated plants | 1,991,580 | 39.67 |
| TR Endosperm | Mature green endosperm dissected from seeds of exposed plants | 5,001,153 | 52.51 |
| CT Progeny | Leaf samples from the progeny of untreated plants | 2,895,793 | 40.84 |
| TR Progeny | Leaf samples from the progeny of exposed plants | 4,067,056 | 33.19 |
| Average |  | 4,873,042 | 52.68 |

**Supplementary table 4. The significantly differentially expressed miRNAs in the pollen of *B. rapa* plants treated with heat shock**

The differentially expressed smRNAs mapped to conservative mature miRNAs (TR vs CT, q<0.2, the Benjamini-Hochberg method). * - denotes a complementary strand of the corresponding mature miRNA.

| **The miRNA gene family** | **Mature differentially expressed miRNAs** | **Log2FC, TR vs CT** | **q-value** | **Length, nt** |
| --- | --- | --- | --- | --- |
| bra-miR158 | bra-miR158a-2 | -2.10 | 1.12E-01 | 19 |
| bra-miR159 | bra-miR159a-1 | 1.24 | 8.64E-04 | 21 |
| bra-miR162 | bra-miR162a | 1.02 | 3.13E-02 | 21 |
| bra-miR166 | bra-miR166a-1 | 1.31 | 1.57E-01 | 19 |
|  | bra-miR166a-1 | 1.68 | 1.82E-08 | 21 |
|  | bra-miR166a-1 | 1.79 | 1.11E-04 | 20 |
|  | bra-miR166a-1 | 2.44 | 4.05E-02 | 21 |
| bra-miR167 | bra-miR167a-1 | 1.19 | 4.86E-02 | 19 |
|  | bra-miR167a-1 | 1.91 | 3.01E-05 | 21 |
|  | bra-miR167a-1* | 1.44 | 5.69E-03 | 21 |
| bra-miR168 | bra-miR168a-1 | 1.18 | 7.47E-02 | 21 |
|  | bra-miR168a-1 | 1.19 | 4.10E-02 | 20 |
|  | bra-miR168a-3 | 1.06 | 1.39E-02 | 21 |
| bra-miR1885 | bra-miR1885a | 1.08 | 6.82E-02 | 22 |
| bra-miR319 | bra-miR319c-1 | 1.47 | 1.56E-02 | 21 |
| bra-miR390 | bra-miR390a-1 | 2.32 | 1.66E-02 | 21 |
|  | bra-miR390a-1* | 1.96 | 1.91E-01 | 20 |
| bra-miR393 | bra-miR393a | 1.41 | 4.51E-02 | 21 |
|  | bra-miR393a | 3.34 | 8.08E-06 | 22 |
| bra-miR395 | bra-miR395a-1 | 1.92 | 7.44E-02 | 21 |
| bra-miR396 | bra-miR396a | 0.83 | 5.56E-02 | 21 |
|  | bra-miR396b | 2.34 | 7.47E-02 | 21 |
| bra-miR398 | bra-miR398b-1 | 2.59 | 1.48E-15 | 21 |
| bra-miR403 | bra-miR403 | 0.79 | 2.89E-02 | 21 |
|  | bra-miR403 | 1.46 | 9.83E-03 | 20 |
| bra-miR408 | bra-miR408a | 1.58 | 9.14E-02 | 20 |
|  | bra-miR408a | 2.02 | 2.16E-03 | 21 |
| bra-miR5718 | bra-miR5718 | 1.32 | 1.91E-04 | 22 |
|  | bra-miR5718* | 1.04 | 1.27E-02 | 21 |
| bra-miR827 | bra-miR827 | 1.02 | 1.90E-01 | 21 |
|  | Predicted miR22711 | -1.34 | 1.95E-01 | 22 |

**Supplementary table 5. The differentially expressed miRNAs in the endosperm of *B. rapa* plants treated with heat shock**

The differentially expressed smRNAs mapped to conservative mature miRNAs (TR vs CT, q<0.2, the Benjamini-Hochberg method). * - denotes a complementary strand of the corresponding mature miRNA.

| **The miRNA gene family** | **Mature differentially expressed miRNAs** | **Log2FC, TR vs CT** | **q-value** | **Length, nt** |
| --- | --- | --- | --- | --- |
| bra-miR167 | bra-miR167a-1 | 4.37 | 0.05 | 20 |
|  | bra-miR167a-2 | 5.63 | 0.04 | 21 |
|  | bra-miR167a-1* | 5.29 | 0.04 | 21 |
| bra-miR168 | bra-miR168a-3 | 6.48 | 0.05 | 21 |
| bra-miR171 | bra-miR171a-1 | 14.89 | 0.06 | 21 |
| bra-miR390 | bra-miR390a-1 | 3.78 | 0.19 | 21 |

**Supplementary table 6. The differentially expressed miRNAs in the progeny of *B. rapa* plants treated with heat shock**

The differentially expressed smRNAs mapped to conservative mature miRNAs (TR vs CT, q<0.2, the Benjamini-Hochberg method). * - denotes a complementary strand of the corresponding mature miRNA.

| **The miRNA gene family** | **Mature differentially expressed miRNAs** |  | **Log2FC, TR vs CT** | **q-value** | **Length, nt** |
| --- | --- | --- | --- | --- | --- |
| bra-miR1140 | bra-miR1140 |  | -2.20 | 1.02E-01 | 20 |
|  | bra-miR1140 |  | -1.80 | 9.64E-02 | 21 |
|  | bra-miR1140* |  | -1.74 | 8.68E-02 | 22 |
| bra-miR165 | bra-miR165a |  | -2.48 | 6.29E-03 | 20 |
|  | bra-miR165a |  | -2.20 | 4.38E-02 | 21 |
|  | bra-miR165a |  | -2.43 | 1.16E-01 | 21 |
| bra-miR166 | bra-miR166c* |  | -2.05 | 5.01E-02 | 21 |
| bra-miR167 | bra-miIR167d |  | -3.29 | 1.35E-01 | 21 |
| bra-miR168 | bra-miR168a-1 |  | -1.57 | 1.58E-01 | 21 |
| bra-miR319 | bra-miR319a-1 |  | -3.74 | 4.13E-07 | 20 |
|  | bra-miR319a-1 |  | -4.45 | 2.73E-10 | 21 |
|  | bra-miR319a-3 |  | -4.32 | 5.43E-02 | 20 |
|  | bra-miR319a-3 |  | -5.79 | 1.85E-08 | 21 |
|  | bra-miR319b* |  | -13.73 | 2.27E-03 | 19 |
|  | bra-miR319c-1 |  | -1.93 | 5.73E-02 | 21 |
| bra-miR390 | bra-miR390a-1 |  | -2.69 | 4.01E-04 | 21 |
|  | Predicted miR31241 |  | -2.18 | 5.99E-02 | 20 |
|  | Predicted miR31241 |  | -1.83 | 7.77E-02 | 21 |
|  | Predicted miR315691 |  | -3.29 | 1.35E-01 | 20 |
|  | Predicted miR315691 |  | -1.67 | 1.62E-01 | 21 |

**Supplementary table 7. The differentially expressed miRNAs in the inflorescence meristem and fertilized ovules of *B. rapa* plants treated with heat shock**

The differentially expressed smRNAs mapped to conservative mature miRNAs (TR vs CT, q<0.2, the Benjamini-Hochberg method).

| **The miRNA gene family** | **Mature differentially expressed miRNAs** | **Log2FC, TR vs CT** | **q-value** | **Length, nt** | **Tissue** |
| --- | --- | --- | --- | --- | --- |
| bra-miR396 | bra-miR396a | -2.50 | 0.03 | 17 | Inflorescence meristem |
|  | bra-miR396a | -2.01 | 0.08 | 17 | Fertilized ovules |

**Supplementary table 8. The expression profile of mature miRNAs of miR168 class in tissues of heat-stressed *B. rapa* parental plants and untreated progeny**

The asterisks denote a significant difference in the expression as compared to controls (the Benjamini-Hochberg method, q<0.2). The positions of miRNA genes were retrieved using the BRAD – *Brassica* Genome Browser v 1.2

| miRNA | Sequence of mature miRNA | Position of the miRNA gene on the chromosome | Tissue | Log2FC, TR vs CT | q-value |
| --- | --- | --- | --- | --- | --- |
| bra-miR168a- 1 | 5΄-TCGCTTGGTGCAGGTCGGGA-3΄ | A01:5386102..  5386238  (+strand)  A03:23663743..  23663871  (+strand)  A06:25071499.. 25071634  (- strand)  A08:10924553..  10924689  (+ strand)  A09:12975267..  12975402  (+ strand)  E-value=0.001 | Leaves | 0.58 | 1 |
|  |  |  | Inflorescence meristem | 0.05 | 1 |
|  |  |  | Pollen | 1.19* | 0.04 |
|  |  |  | Unfertilized ovules | -0.54 | 1 |
|  |  |  | Fertilized ovules | -0.62 | 1 |
|  |  |  | Embryo | 0.61 | 1 |
|  |  |  | Endosperm | 3.42* | 0.41 |
|  |  |  | Progeny | -0.58* | 0.93 |
| bra-miR168a- 2 | 5΄-TCGCTTGGTGCAGGTCGGGAC-3΄ | A01:5386102..  5386238  (+strand)  A03:23663743..  23663871  (+ strand)  E-value =4E-04 | Leaves | 0.61 | 1 |
|  |  |  | Inflorescence meristem | 0.06 | 1 |
|  |  |  | Pollen | 1.18* | 0.07 |
|  |  |  | Unfertilized ovules | -0.88 | 1 |
|  |  |  | Fertilized ovules | -0.89 | 1 |
|  |  |  | Embryo | 0.51 | 1 |
|  |  |  | Endosperm | 3.37* | 0.29 |
|  |  |  | Progeny | -1.57* | 0.16 |
| bra-miR168a- 3 | 5΄-TCGCTTGGTGCAGGTCGGGAA-3΄ | A06:25071499.. 25071634  (- strand)  A08:10924553..  10924689  (+ strand)  A09:12975267..  12975402  (+ strand)  E-value =  4E-04 | Leaves | 0.51 | 1 |
|  |  |  | Inflorescence meristem | 0.30 | 1 |
|  |  |  | Pollen | 1.06* | 0.01 |
|  |  |  | Unfertilized ovules | -0.81 | 1 |
|  |  |  | Fertilized ovules | -0.42 | 1 |
|  |  |  | Embryo | 0.56 | 1 |
|  |  |  | Endosperm | 6.48* | 0.05 |
|  |  |  | Progeny | -0.71* | 0.85 |

**Supplementary table 9. Primers and probes used in this study**

| **Primer/Probe Name** | **Sequence** | **Description** |
| --- | --- | --- |
| AB492 | 5΄-DigN-TCCCGACCTGCACCAAGCGA-DigN-3΄ | DIG labelled probe for northern blot detection of miR168 |
| AB445 | 5΄-TTGGAATTGTCGAGGGACTC-3΄ | Forward qPCR primer – *braGAPDH*-AF536826 |
| AB446 | 5΄-GAGCTGTGGAAGCACCTTTC-3΄ | Reverse qPCR primer – *braGAPDH*-AF536826 |
| AB447 | 5΄-CTCGATGGCCTCAACCTTTA-3΄ | Forward qPCR primer – *braTUBULINE* - D78496 |
| AB448 | 5΄-ATGTTGCTCTCGGCTTCTGT-3΄ | Reverse qPCR primer – *braTUBULINE* - D78496 |
| AB449 | 5΄-GAGCATACCGGTCTCCACAC-3΄ | Forward qPCR primer – *braEF1a* - GO479260 |
| AB450 | 5΄-AAAGAGGCCATCAGACAAGC-3΄ | Reverse qPCR primer – *braEF1a* - GO479260 |
| AB451 | 5΄-TAACTGCGACTCAGGGAATCTT-3΄ | Forward qPCR primer – *braUBC* - GO479262 |
| AB452 | 5΄-TCATCCTTTCTTAGGCATAGCG-3΄ | Reverse qPCR primer – *braUBC* - GO479262 |
| AB493 | 5΄-TCATGATTCTGGGAGGGAAG-3΄ | Forward qPCR primer – *braAGO1* - Bra032254 |
| AB494 | 5΄-TGGCACATCTGAGCAAGTTC-3΄ | Reverse qPCR primer – *braAGO1* - Bra032254 |

**Supplementary figure 1. Experimental setup**

Two groups were obtained for the experiment – “Control” and “Heat shock”. Thirty *B. rapa* plants per group were used, and every tissue was harvested in duplicates. Dpg – days post germination. F0 – parental plants, F1 - progeny

**Supplementary figure 2. Gene ontology annotation of differentially expressed genes in the selected *B. rapa* tissues**

**A** – Leaves, **B** – Inflorescence meristem, **C** – Unfertilized ovules, **D** – Fertilized ovules. Coding sequences of differentially expressed genes (TR vs CT) were extracted from the *B. rapa* transcriptome database v 1.2 and loaded as a FASTA file into Blast2GO v 2.6.2 software for the NCBI BLAST similarity search using blastx option (Conesa, Gotz et al. 2005). Further, the recovered ontologies were annotated and grouped into gene ontology categories using default settings. Gene ontology (GO) nodes were combined into the most prominent categories using a GO-slim-TAIR tool and represented as the percentage of up- and down-regulated genes in the corresponding GO category.

**Supplementary figure 3. Gene ontology annotation of differentially expressed genes in the selected *B. rapa* tissues**

**A** – Embryo, **B** – Endosperm, **C** – Progeny. Coding sequences of differentially expressed genes (TR vs CT) were extracted from the *B. rapa* transcriptome database v 1.2 and loaded as a FASTA file into Blast2GO v 2.6.2 software for the NCBI BLAST similarity search using blastx option (Conesa, Gotz et al. 2005). Further, the recovered ontologies were annotated and grouped into gene ontology categories using default settings. Gene ontology (GO) nodes were combined into the most prominent categories using a GO-slim-TAIR tool and represented as the percentage of up- and down-regulated genes in the corresponding GO category.

**Supplementary figure 4. The relative length distribution of sequencing reads from the total small RNA library of the corresponding tissue**

**A** – Leaves, **B** – Inflorescence meristem, **C** – Pollen, **D** – Unfertilized ovules, **E** – Fertilized ovules, **F** – Embryo, **G** – Endosperm, **H** – Progeny. The values represent the mean ± SD of the relative enrichment of smRNA fraction in the total library of the corresponding tissue. The asterisk shows a statistically significant difference as compared to the control library of the corresponding tissue (Student’s t-test: α=0.05, t=2.45).

**Supplementary figure 5. The relative length distribution of sequencing reads mapped to gene regions in the total small RNA library of the corresponding tissue**

**A** – Leaves, **B** – Inflorescence meristem, **C** – Pollen, **D** – Unfertilized ovules, **E** – Fertilized ovules, **F** – Embryo, **G** – Endosperm, **H** – Progeny. The values represent the mean ± SD of the relative enrichment of smRNA fraction in the total library of the corresponding tissue.

**Supplementary figure 6. The relative length distribution of sequencing reads mapped to transposon element regions in the total small RNA library of the corresponding tissue**

**A** – Leaves, **B** – Inflorescence meristem, **C** – Pollen, **D** – Unfertilized ovules, **E** – Fertilized ovules, **F** – Embryo, **G** – Endosperm, **H** – Progeny. The values represent the mean ± SD of the relative enrichment of smRNA fraction in the total library of the corresponding tissue. The asterisks show a statistically significant difference as compared to the control library of the corresponding tissue (Student’s t-test: α=0.05, t=2.45).

**Supplementary figure 7. Small RNA Northern blot of bra-miR168 in heat-shock-stressed *B. rapa* parental tissues and untreated progeny**

The total RNA was used as a loading control. The probe was designed to hybridize to the consensus sequence of mature bra-miR168miRNAs.
