# Supplementary figures and images for: The elucidation of stress memory inheritance in Brassica rapa plants
Source: Front Plant Sci. 2015 Jan 21;6:5. doi: 10.3389/fpls.2015.00005 (PMC4300914; doi:10.3389/fpls.2015.00005)

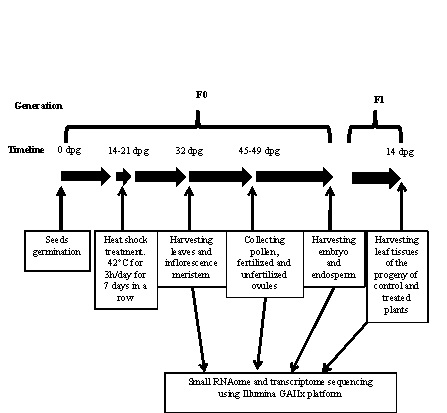

Supplement: Supplementary file 2 [file Image1.JPEG]

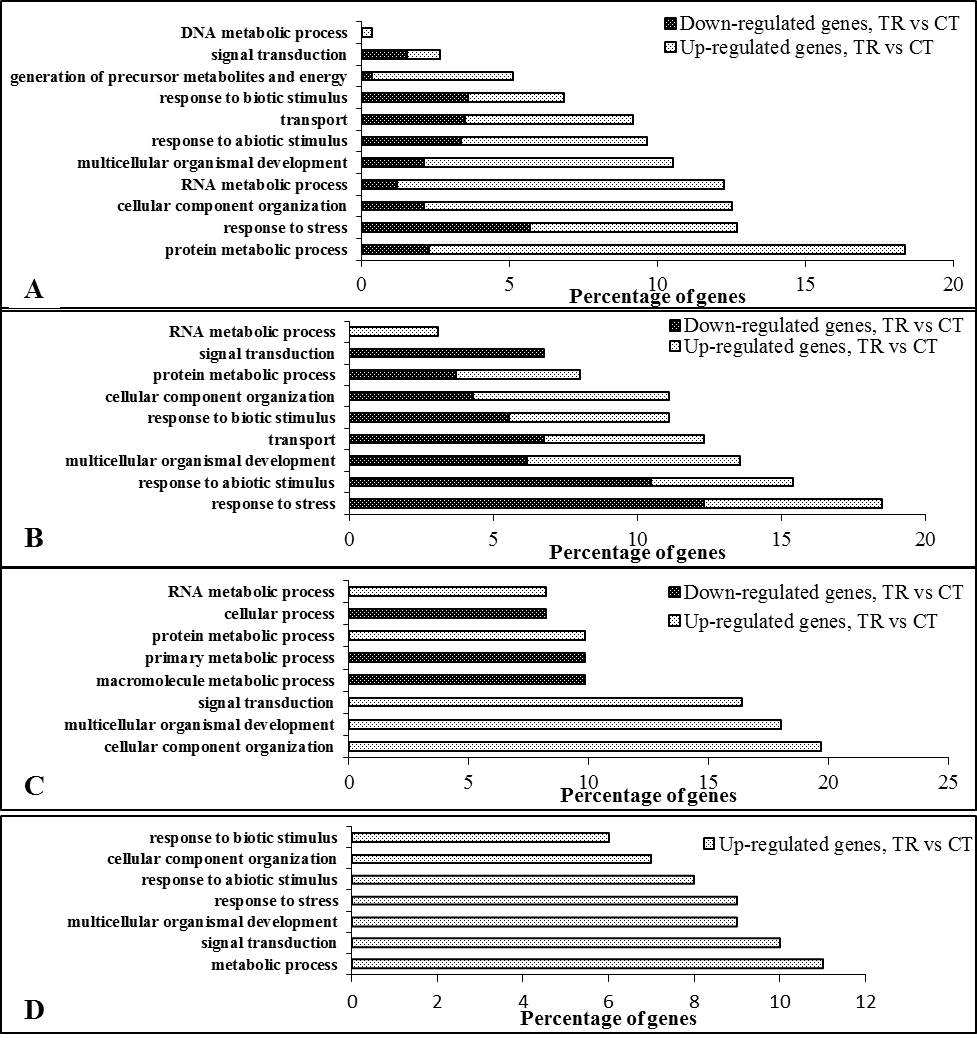

Supplement: Supplementary file 3 [file Image2.JPEG]

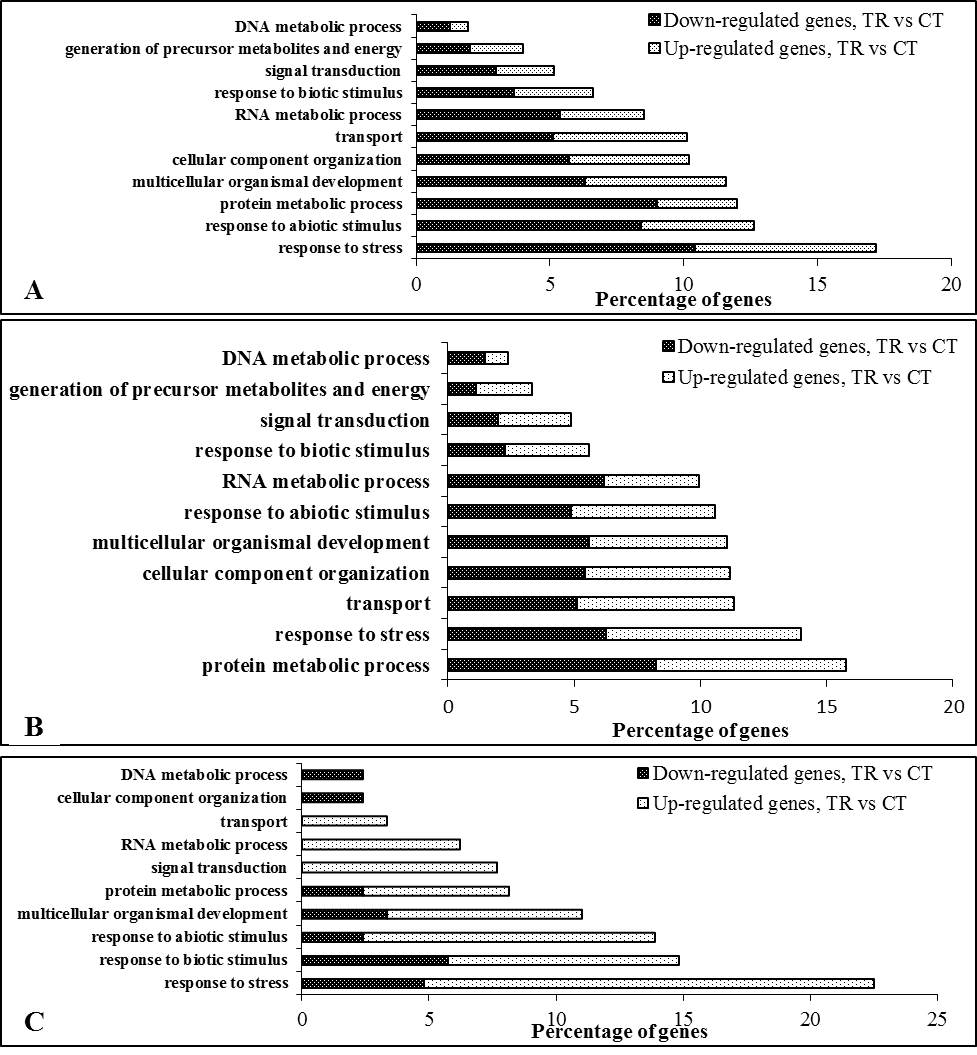

Supplement: Supplementary file 4 [file Image3.JPEG]

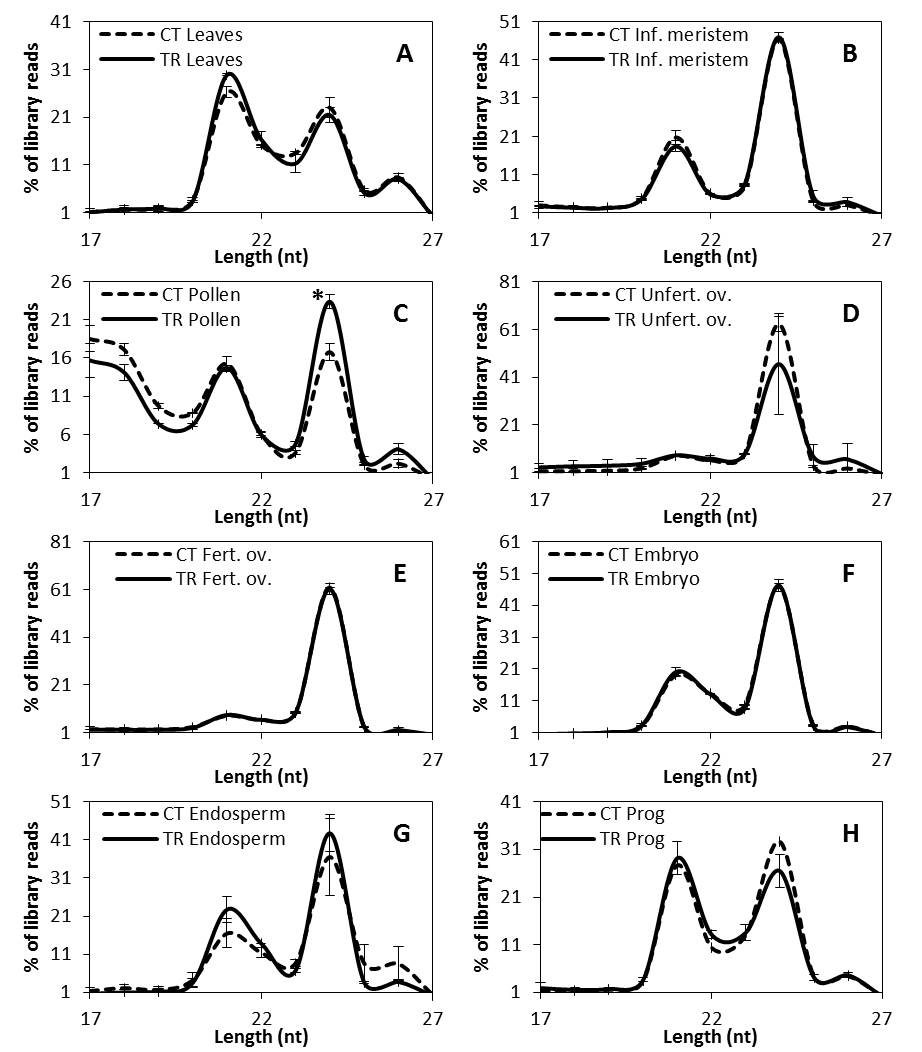

Supplement: Supplementary file 5 [file Image4.JPEG]

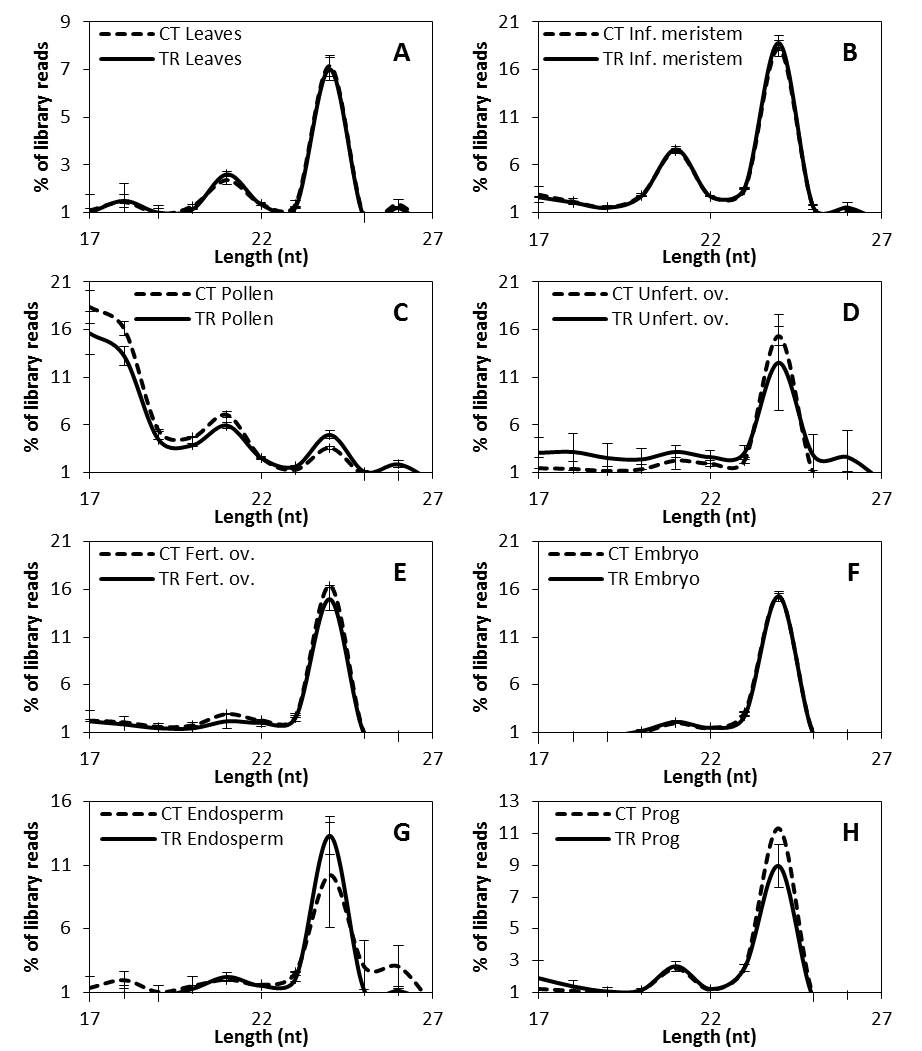

Supplement: Supplementary file 6 [file Image5.JPEG]

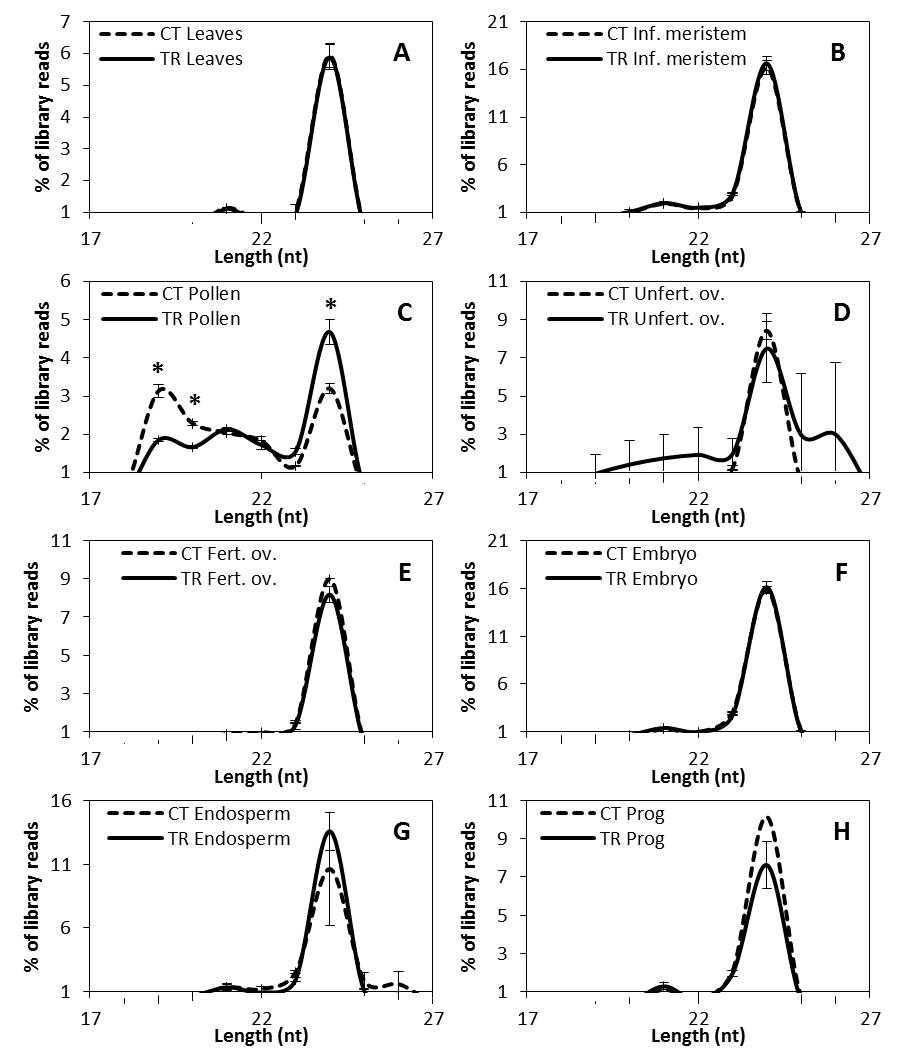

Supplement: Supplementary file 7 [file Image6.JPEG]

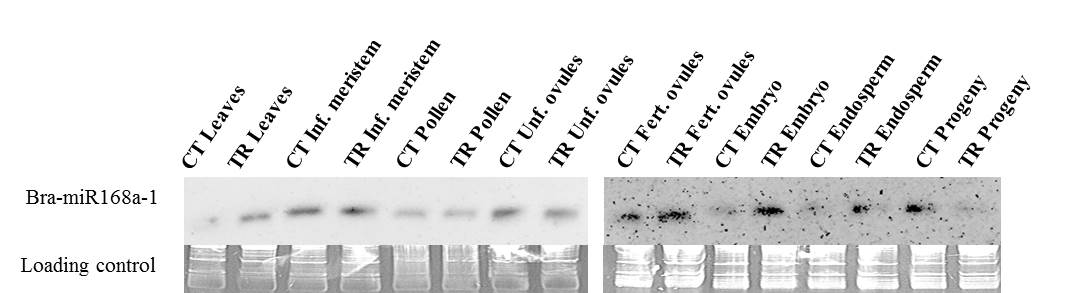

Supplement: Supplementary file 8 [file Image7.JPEG]
